# Global epidemiology of type 1 diabetes in young adults and adults: a systematic review

**DOI:** 10.1186/s12889-015-1591-y

**Published:** 2015-03-17

**Authors:** Paula A Diaz-Valencia, Pierre Bougnères, Alain-Jacques Valleron

**Affiliations:** Institut National de la Santé et de la Recherche Médicale, Inserm U-1169, F-94276 Kremlin Bicêtre Paris, France; Pierre et Marie Curie University, Paris, France; Paris-Sud University, Paris, France

**Keywords:** Type 1 diabetes, Systematic review, Adults, Incidence, Epidemiology

## Abstract

**Background:**

Although type 1 diabetes (T1D) can affect patients of all ages, most epidemiological studies of T1D focus on disease forms with clinical diagnosis during childhood and adolescence. Clinically, adult T1D is difficult to discriminate from certain forms of Type 2 Diabetes (T2D) and from Latent Autoimmune Diabetes in Adults (LADA).

We searched the information available worldwide on the incidence of T1D among individuals over 15 years of age, and which diagnostic criteria should be used use to qualify T1D in adults. We then studied the variation of T1D incidence with age in adults, and compared it to the incidence in the <15 years-old.

**Methods:**

A systematic review of the literature was performed to retrieve original papers in English, French and Spanish published up to November 6, 2014, reporting the incidence of T1D among individuals aged over 15 years. The study was carried out according to the PRISMA recommendations.

**Results:**

We retrieved information reporting incidence of T1D among individuals aged more than 15 years in 35 countries, and published in 70 articles between 1982 and 2014. Specific anti-beta-cell proteins or C-peptide detection were performed in 14 of 70 articles (20%). The most frequent diagnostic criteria used were clinical symptoms and immediate insulin therapy. Country-to-country variations of incidence in those aged >15 years paralleled those of children in all age groups. T1D incidence was larger in males than in females in 44 of the 54 (81%) studies reporting incidence by sex in people >15 years of age. The overall mean male-to-female ratio in the review was 1.47 (95% CI = 1.33-1.60, SD = 0.49, n = 54, p = <0.0001). Overall, T1D incidence decreased in adulthood, after the age of 14 years.

**Conclusions:**

Few studies on epidemiology of T1D in adults are available worldwide, as compared to those reporting on children with T1D. The geographical variations of T1D incidence in adults parallel those reported in children. As opposed to what is known in children, the incidence is generally larger in males than in females. There is an unmet need to evaluate the incidence of autoimmune T1D in adults, using specific autoantibody detection, and to better analyze epidemiological specificities – if any – of adult T1D.

**PROSPERO registration number:**

CRD42012002369.

**Electronic supplementary material:**

The online version of this article (doi:10.1186/s12889-015-1591-y) contains supplementary material, which is available to authorized users.

## Background

The worldwide epidemiology of childhood Type 1 diabetes (T1D) was extensively described in the 6th edition of the International Diabetes Federation (IDF) [1]. Data were retrieved in approximately 45% of the countries [1-4]. In contrast, we are unaware of a similar review on the worldwide epidemiology of adult T1D diabetes, although T1D is known to occur even late in adults [5-7]. A major limitation of the epidemiology of T1D in adults is certainly the difficulty there is to distinguish it from Type 2 diabetes (T2D) requiring insulin treatment or from Latent Autoimmune Diabetes in Adults (LADA), when specific markers of autoimmunity are not searched.

Here, our primary objective was to describe – through a systematic review of the literature – the available published information on adult T1D incidence, and the diagnostic criteria used for case definition. A secondary objective was to study how the variations of T1D incidence in adults mirrored those in children.

## Methods

### Literature review

A systematic review was conducted according to the PRISMA recommendations to retrieve original papers published in English, French and Spanish up to November 6th, 2014, in peer-reviewed journals reporting the incidence of T1D among individuals aged more than 15 years, in population-based studies (*i.e.* collected in a defined geographic area [8]) and reporting the diagnostic criteria used to define T1D.

**Figure 1:**
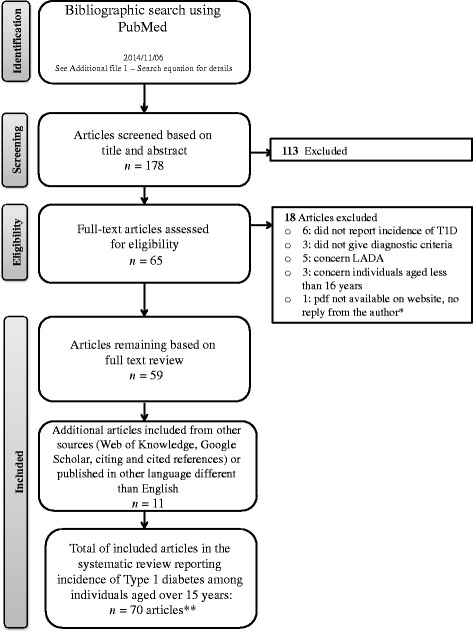
**PRISMA Flow diagram bibliographic search strategies.** * Kumar P, et al. Indian Med Assoc. 2008;106(11):708–711. ** The article: Radosevic B, et al. Pediatr Diabetes. 2013;14(4):273–4 gives information from two countries: 1) Bosnia and Herzegovina: Republic of Srpska and 2) Slovenia, Nationwide.

The databases used for the literature search were Medline (PubMed), Google Scholar and Thomson Reuters (Web of Knowledge). The protocol of the search was registered in the International Prospective Register of Systematic Reviews (PROSPERO) and is available on http://www.crd.york.ac.uk/PROSPERO/display_record.asp?ID=CRD42012002369 (Registration number: 2012:CRD42012002369). Figure 1 presents the flow diagram of the bibliographic search, Additional file 1 for the full electronic search strategy, and Additional file 2 for the PRISMA checklist.

### Data collection

For each study, the following information was extracted:

- the identification of the study: authors, title, journal, publication year,
- the period and country of study. The country was categorized by its World Health Organization (WHO) region and economic level: high-income (HIGH) or low- and middle-income (LMIC) [9],
- the geographic coverage of the study: nationwide (when the study was performed in the entire nation) and local (when it was restricted to a given region, city, or a geographically defined population),
- the diagnostic criteria used to define T1D in adults: detection of autoantibodies against beta-cells (such as: islet cell antibody (ICA), insulin autoantibody (IAA), islet antigen-2 autoantibody (IA-2), anti-glutamic acid decarboxylase antibodies (GAD)), measurement of the fasting C-peptide level [7], need for permanent insulin therapy, time when the administration of insulin therapy was started, and clinical signals of T1D diabetes such as ketosis, ketonuria and weight loss,
- the sources of data/registers reporting T1D incidence in the studies, defined according to LaPorte et al. [10] as: *primary* source of information: a “well-established system of standardized registries for identifying new cases”, for example national or regional registers, *secondary* source of information: other different sources of cases “that would provide a check on the degree of ascertainment”, for example medical records or hospital discharges, and *tertiary* source of information: a third approach for identifying cases, for example, through surveillance system or death certificates,
- the reported percentage of completeness/ascertainment between sources of information reporting incidence [10],
- the incidence rates reported in the text, tables or graph (expressed as new cases per 100.000 persons/year) by sex and age classes,
- additional information such as those concerning rural/urban, or ethnic differences.

### Data analyses

The country distribution of the T1D incidence information and the analysis of the diagnostic criteria used were performed on the entire set of articles retrieved. For the few papers for which the results were presented by ethnic origin, we estimated the mean value of the incidence for the given period in the countries/regions concerned.

### Correlation between adult and children T1D incidences

In the geographical correlation analyses between children and adult incidences, we considered for each country the more recent nationwide study published, or if not available, the last published set of local studies retrieved from a given area in the country; in addition, we included all published papers reporting auto-antibodies against beta-cells or C-peptide. To obtain an estimate of the incidence of T1D in children in the countries for which the adult incidence was available, we used the data provided by the same adult paper, when available. The incidence of T1D in children was not available in 9 of these papers included in the geographical correlation analyses. In this case, it was estimated through a separate systematic review focused on the corresponding countries and periods (see Additional file 3).

### Statistics

Data were extracted from graphs using GraphClick [11].

The country-to-country co-variation of children and adult incidences was quantified by the Spearman correlation and a linear regression.

The R software (version 3.0.1) was used for statistical and graphic analyses [12].

## Results

### Description of the information obtained from the systematic review on adult T1D

Seventy articles reporting incidence of T1D in young adults and adults aged over than 15 years concerned one country, and one article concerning two countries were retrieved in this systematic review, resulting in a total of 71 studies covering 35 countries (Table 1). Twenty-four of the 71 studies were nationwide; 43 papers provided information on the T1D incidence in the age class 15–29 years, 26 in the age class 30–59 years, and 6 in the persons aged >60 years.

**Table 1:** **Systematic review of T1D in adults, diagnostic criteria and sources of information**

**Study information**					**T1D diagnosis criteria in adults and young adults**						**Source of information and validation of ascertainment between sources**			
**Country, area reported in the article**	**First author, publication year**	**Ref**	**Age range**	**Period**	**Detect. AA/C-Peptide**	**Need of insulin therapy**	**Administration insulin therapy**	**Clinical impression**	**Ketosis/ ketonuria**	**Weight loss**	**Primary**	**Secondary**	**Tertiary**	**% of ascertainment**
**African Region, LMIC**														
Mauritius: NW	Tuomileht J., 1993^†^	[13]	0-19	1986-1990	No	Yes	From diagnosis	Yes	NA	NA	Medical reports	Medical statistics	NA	95.0
United Republic of Tanzania: Dar es Salaam	Swai A. B., 1993^†^	[14]	0-19	1982-1991	No	Yes	From diagnosis	Yes	NA	NA	Medical reports	Hospital records	NA	NA
**Eastern Mediterranean Region, LMIC**														
Iran (Islamic Republic of): Fars	**Pishdad G. R., 2005** ^**†**^	[15]	0-29	1990-1994	**Yes (a)**	Yes	From diagnosis	Yes	Yes	Yes	Medical reports from endocrinologists	Medical records	NA	100
Libyan Arab Jamahiriya: Benghazi	Kadiki O. A., 1996^†^	[16]	0-34	1981-1990	No	Yes	From diagnosis	NA	Yes	NA	National Diabetes Program	Hospital registers	NA	95.0
Tunisia: Beja, Monastir, Gafsa	Ben Khalifa F., 1998^†^	[17]	0-19	1990-1994	No	Yes	From diagnosis	Yes	NA	NA	Hospital records	School health centers	NA	96.0
**European Region, LMIC**														
Croatia: Zagreb	Roglic G., 1995^†^	[18]	0- > 55	1988-1992	No	Yes	Within 1 week of diagnosis	Yes	Yes	NA	National Diabetes Program	Death certificates	Diabetes association	96.2
Estonia: NW	Kalits I., 1990^†^	[19]	0- > 50	1988-1988	No	Yes	From diagnosis	Yes	Yes	Yes	NA	NA	NA	NA
Lithuania: NW	Ostrauskas R., 2011^†^	[20]	15-34	1991-2008	No	Yes	Within 2 weeks of diagnosis	Yes	Yes	Yes	National Diabetes Program	Regional endocrinologist	Notes of patient insurance	86.8
Lithuania: NW	Pundziute-Lycka A., 2003	[21]	0-39	1991-2000	No	Yes	Within 2 weeks of diagnosis	Yes	Yes	NA	National Diabetes Program	Pediatrician and endocrinologist reports	Death certificates	91.2
Lithuania: NW	Ostrauskas R., 2000	[22]	15-39	1991-1997	No	Yes	Within 2 weeks of diagnosis	Yes	Yes	NA	National Diabetes Program	Pediatrician and endocrinologist reports	Death certificates	91.2
Poland: Bialystok	Kretowski A., 2001^†^	[23]	0-29	1994-1998	No	Yes	From diagnosis	Yes	Yes	Yes	Pediatric and Internal medicine records	Hospital discharge registers	NA	98.5
Poland: Province of Rzeszow	Sobel-Maruniak A., 2006^†^	[24]	0-29	1980-1999	No	Yes	From diagnosis	Yes	NA	NA	Pediatric and Internal medicine records	Others health care registers	NA	99.0
Poland: Province of Rzeszow	Grzywa M. A., 1995	[25]	0-29	1980-1992	No	Yes	From diagnosis	Yes	NA	NA	Pediatric and Internal medicine records	Others health care registers	NA	99.0
Poland: Warsaw	Wysock M. J., 1992^†^	[26]	0-29	1983-1988	No	Yes	From diagnosis	Yes	NA	NA	Medical records from diabetic clinics	General practitioners and diabetologist registers	Death certificates	NA
Romania: Bucharest	Ionescu-Tirgoviste C., 1994^†^	[27]	0- ≥ 85	1981-1991	No	Yes	From diagnosis	Yes	Yes	NA	Bucharest Diabetes Registry	NA	NA	NA
Slovakia: NW	Kyvik K O, 2004^†^	[28]	15-29	1996-1997	No	Yes	From diagnosis	Yes	NA	NA	Pediatrician and endocrinologist reports	Other health care registers	NA	80.0
**European Region, HIGH**														
Austria: Upper	Rami B., 2001^†^	[29]	0-29	1994-1996	No	Yes	From diagnosis	Yes	NA	NA	Pediatricians and endocrinologists reports	Austrian Diabetes Association	NA	87.0
Belgium: Antwerp	**Weets I**., **2007** ^**†**^	[30]	0-39	1989-2003	**Yes**	Yes	From diagnosis	Yes	NA	NA	Pediatricians and endocrinologists reports	General practitioners and diabetes nurses reports	Diabetes associations and self-reporting	97.0
Belgium: Antwerp	**Weets I., 2002** ^**†**^	[31]	0-39	1989-2000	**Yes**	Yes	From diagnosis	NA	NA	NA	Pediatrician and endocrinologist reports	General practitioner and diabetes nurse reports	Diabetes associations and self-reporting	93
Belgium: Antwerp	**Vandewalle C., 1997** ^**†**^	[32]	0-39	1989-1995	**Yes**	Yes	From diagnosis	Yes	Yes	Yes	Pediatrician and endocrinologist reports	General practitioner and diabetes nurse reports	Diabetes associations and self-reporting	85
Bosnia and Herzegovina: Republic of Srpska	Radosevic B., 2013^†^	[33]	0-18	1998-2010	No	Yes	From diagnosis	Yes	NA	NA	Hospital records	Insulin prescription registers	NA	100
Denmark: Copenhagen and Frederiksborg	**Molbak A. G**., **1994** ^**†**^	[34]	30-95	1973-1977	**Yes (b)**	Yes	From diagnosis	Yes	Yes	Yes	Hospital discharges	General practitioners and diabetologist registers and death certificates	Missing coding of T1D diagnosis in hospital admissions	99.0
Finland: NW	**Lammi N**., **2007** ^**†**^	[35]	15-39	1992-1996	**Yes**	Yes	From diagnosis	Yes	NA	NA	National Diabetes Program	Hospital discharge registers	Drug reimbursement registers	88.0
France: Aquitaine, Lorraine, Basse Normandie, Haute Normandie	Charkaluk M. L, 2002^†^	[36]	0-19	1988-1997	No	Yes	None declared	NA	NA	NA	Prospective registers	French Social Security registers	NA	96.0
France: Aquitaine, Lorraine, Basse Normandie, Haute Normandie	Levy-Marchal, C., 1998	[37]	0-19	1988-1995	No	Yes	None declared	NA	NA	NA	Prospective registers	French Social Security registers	NA	96.0
Israel: NW	Blumenfeld O., 2014^†^	[38]	0-17	1997-2010	No	Yes	From diagnosis	Yes	NA	NA	Israel juvenile diabetes register	Israel Center for Disease Control	NA	NA
Israel: NW	Sella T., 2011	[39]	0-17	2000-2008	No	Yes	None declared	Yes	NA	NA	Israel juvenile diabetes register	Israel Center for Disease Control	NA	NA
Israel: NW	Koton S., 2007	[40]	0-17	1997-2003	No	Yes	From diagnosis	Yes	NA	NA	Israel juvenile diabetes register	NA	NA	NA
Italy: Lombardie	Garancini, P., 1991†	[41]	0-34	1981-1982	No	Yes	None declared	NA	NA	NA	Hospital discharge records	Hospital admission records	NA	85.7
Italy: Pavia	Tenconi M. T., 1995^†^	[42]	0-29	1988-1992	No	Yes	From diagnosis	Yes	NA	NA	Hospital records	Drug registers	NA	100
Italy: Sardinia	Muntoni S, 1992^†^	[43]	0-29	1989-1990	No	Yes	From diagnosis	Yes	NA	NA	Hospital records	Diabetes association	NA	92.8
Italy: Sardinia (Oristano)	Frongia O., 1997^†^	[44]	0-29	1993-1996	No	Yes	From diagnosis	Yes	NA	NA	Hospital records	Drug registers	NA	100
Italy: Turin	**Bruno G**., **2009** ^**†**^	[45]	15-29	2000-2004	**Yes**	Yes	Within 6 months of diagnosis	NA	NA	NA	Hospital records	Drug registers	NA	NA
Italy: Turin	**Bruno G**., **2005** ^**†**^	[46]	30-49	1999-2001	**Yes**	Yes	Within 6 months of diagnosis	NA	Yes	NA	Diabetes clinics	Drug registers	NA	99.0
Italy: Turin	Bruno G., 1993	[47]	0-29	1984-1988	No	Yes	From diagnosis	NA	Yes	NA	Diabetic clinics records	Hospital discharge records	NA	97.0
Luxembourg: NW	De Beaufort C. E., 1988^†^	[48]	0-19	1977-1986	No	Yes	None declared	NA	NA	NA	Pediatric and Internal medicine records	Dutch Diabetes Association	NA	100
Malta: NW	Schranz A. G., 1989^†^	[49]	0-24	1980-1987	No	Yes	Within 3 moths of diagnosis	Yes	Yes	Yes	Medical reports	Diabetic clinic records	NA	NA
Netherlands: NW	Ruwaard D., 1994^†^	[50]	0-19	1988-1990	No	Yes	None declared	NA	NA	NA	Pediatric and Internal medicine records	NA	NA	81.0
Norway: NW	Joner G., 1991^†^	[51]	15-29	1978-1982	No	Yes	From diagnosis	NA	NA	NA	Pediatricians and endocrinologists reports	Hospital records	NA	90.0
Slovenia: NW	Radosevic B., 2013^†^	[33]	0-18	1998-2010	No	Yes	From diagnosis	Yes	NA	NA	Slovenian National Registry of Childhood diabetes	Insulin prescription registers	NA	100
Spain: Badajoz	Morales-Perez F. M., 2000^†^	[52]	0-29	1992-1996	No	Yes	From diagnosis	Yes	Yes	NA	Pediatricians and endocrinologists reports	Diabetic clinic records	NA	95.0
Spain: Canarias Islands	Carrillo Dominguez, A., 2000^†^	[53]	0-30	1995-1996	No	Yes	None declared	Yes	NA	Yes	Hospital records and Endocrinologist reports	Diabetes association reports and sales on blood glucose monitors	NA	90.1
Spain: Catalonia	**Abellana R**., **2009** ^**†**^	[54]	0-29	1989-1998	**Yes (c)**	Yes	From diagnosis	Yes	Yes	NA	Catalan Registry of Type 1 Diabetes	Summer camps, associations, and prescription data	NA	90.0
Spain: Catalonia	Goday A., 1992	[55]	0-29	1987-1990	No	Yes	From diagnosis	Yes	NA	NA	Catalan Registry of Type 1 Diabetes	Summer camps, patient associations, and prescription data	NA	90.1
Spain: Navarra	**Forga L**., **2014** ^**†**^	[56]	0- > 45	2009-2012	**Yes**	Yes	Within 6 months of diagnosis	Yes	Yes	NA	Hospital records	Electronic medical records, diabetes associations	NA	98.4
Spain: Navarra	**Forga L**., **2013** ^**†**^	[57]	0-79	2009-2011	**Yes**	Yes	Within 6 months of diagnosis	Yes	Yes	NA	Hospital records	Electronic medical records, diabetes associations	NA	98.4
Sweden: NW	Dahlquist G. G., 2011^†^	[58]	0-34	1983-2007	No	Yes	From diagnosis	Yes	Yes	Yes	National Diabetes Program	Pediatricians and endocrinologist reports	NA	96.0
Sweden: NW	Östman J., 2008	[59]	15-34	1983-2002	No	Yes	From diagnosis	Yes	NA	NA	National Diabetes Program	Pediatrician and endocrinologist reports	Computer-based patient administrative register	82
Sweden: NW	Pundziute-Lycka A., 2002	[60]	0-34	1983-1998	No	Yes	From diagnosis	Yes	Yes	Yes	National Diabetes Program	Pediatrician and endocrinologist reports	Computer-based patient administrative register	91.2
Sweden: NW	Nyström L., 1992	[61]	0-34	1983-1987	No	Yes	None declared	NA	NA	NA	National Diabetes Program	Hospital admission and discharge registers	NA	89
Sweden: NW	Blohme G., 1992	[62]	15-34	1983-1987	No	Yes	From diagnosis	Yes	Yes	Yes	National Diabetes Program	Hospital admission and discharge registers	NA	NA
Sweden: Kronoberg	**Thunander M**., **2008** ^**†**^	[63]	0-100	1998-2001	**Yes**	Yes	Within 4 weeks of diagnosis	Yes	Yes	NA	Opportunistic screening of all adult patients in contact with the medical care system	Departments of ophthalmology	NA	98.0
United Kingdom: NW	Imkampe A. K., 2011^†^	[64]	0-34	1991-2008	No	Yes	Within 3 moths of diagnosis	Yes	NA	NA	National Diabetes Program	Pediatricians and endocrinologist reports	NA	NA
United Kingdom: Oxford region	Bingley P. J., 1989	[65]	0-21	1985-1986	No	Yes	From diagnosis	Yes	NA	NA	Medical reports from general practioners and pediatricians	Regional hospital records	NA	95.0
**Region of the Americas, LMIC**														
Barbados: NW	Jordan O. W., 1994^†^	[66]	0-29	1982-1991	No	Yes	From diagnosis	Yes	NA	NA	Hospital records	Others health care registers	NA	94.0
**Region of the Americas, HIGH**														
Canada: Quebec	Legault L., 2006^†^	[67]	0-18	2000	No	Yes	None declared	NA	NA	NA	Departmental program: Régie des Rentes du Québec program	NA	NA	NA
United States of America: Alabama (Jefferson County)	Wagenknecht L. E., 1991^†^	[68]	0-19	1979-1988	No	Yes	None declared	NA	NA	NA	Hospital records	Summer camps, patient associations, and prescription data	NA	NA
United States of America: Alabama (Jefferson County)	Wagenknecht L. E.,1989	[69]	0-19	1979-1985	No	Yes	From diagnosis	Yes	NA	NA	Hospital records	Association registers	NA	95.0
United States of America: Colorado	Vehik K., 2007^†^	[70]	0-17	2000-2004	No	Yes	Within 2 weeks of diagnosis	Yes	NA	NA	Pediatricians and endocrinologists reports	Other health care registers	The SEARCH Study	96.5
United States of America: Colorado	Kostraba J. N., 1992	[71]	0-17	1978-1988	No	Yes	Within 2 weeks of diagnosis	Yes	NA	NA	Pediatricians and endocrinologists reports	Hospital registers	NA	93.3
United States of America: Pennsylvania (Allegheny)	Libman I. M., 1998^†^	[72]	0-19	1990-1994	No	Yes	From diagnosis	Yes	NA	NA	Medical reports	General practitioners and diabetes nurses reports	NA	97.7
United States of America: Rhode Island	Fishbein H. A., 1982^†^	[73]	0-29	1979-1980	No	Yes	None declared	NA	NA	NA	Medical reports	Insulin prescription registers	NA	NA
United States of America: five areas ^§^	**Bell R., 2009** ^**†**^	[74]	0-19	2002-2005	**Yes**	Yes	From diagnosis	Yes	NA	NA	Medical reports	Other health care registers	The SEARCH Study	NA
United States of America: Wisconsin	Allen C., 1986^†^	[75]	0-29	1970-1979	No	Yes	From diagnosis	Yes	NA	NA	Hospital discharges	Pediatricians and endocrinologist reports	NA	90.0
United States of America: The United States Navy	Gorham C., 1993	[76]	17-34	1974-1988	No	NA*	None declared	Yes	NA	NA	Hospital discharges	NA	NA	NA
**Western Pacific Region, HIGH**														
Australia: New South Wales	Tran F., 2014^†^	[77]	10-18	2001-2008	No	Yes	NA	Yes	NA	Yes	Endocrine group diabetes register	National diabetes register	NA	96.0
Australia: Sydney (Southern Metropolitan Health Region)	Sutton L., 1989^†^	[78]	0-19	1984-1987	No	Yes	From diagnosis	Yes	NA	NA	Medical reports from general practioners and pediatricians	Schools in the area	Syringe register	NA
Japan: Osaka	Sasaki A., 1992^†^	[79]	0-18	1978-1988	No	Yes	None declared	Yes	Yes	NA	Medical benefits system	NA	NA	NA
New Zealand: Canterbury	Scott, R. S., 1991^†^	[80]	0- ≥ 80	1981-1986	No	Yes	Within 1 year of diagnosis	Yes	NA	Yes	Community-based surveys administrated in pharmacies where diabetic patients acquired their insulin supplies	Hospital admission and discharge registers and diabetologist	NA	95.0
**Other Regions currently non WHO**														
Taiwan: NW	**Lin W.-H., 2013** ^**†**^	[81]	0- ≥ 60	1999-2010	**Yes**	Yes	None declared	Yes	Yes	NA	National Health Insure register and Illness certificates	Random sample of a database used to reimbursements	NA	98.3
US Virgin Islands: NW	Washington R. E., 2013^†^	[82]	0-19	2001-2010	No	Yes	From diagnosis	Yes	Yes	Yes	Medical reports	Medical providers	NA	98.7

WHO Member States are divided into high-income (HIGH) or low- and middle-income (LMIC) states [30]. AA: autoantibodies, NW: Nation-wide study, NA: Unavailable data. (a) When there were diagnostic doubts, (b) Only for patients aged over 40 years at onset, (c) Not performed in all cases; the author of this study was contacted to confirm the proportion of these cases, but by the time of submission of this paper no answer was available. T1D: Type 1 Diabetes. Highlighted: reports of the systematic review using the autoantibodies/C-peptide as diagnosis criteria. (^†^) Studies used in the statistical analyses. (*) Data were not available but researchers assumed that patients have had T1D based on their average of age. (^§^) Ohio (8 counties), Washington State (5 counties), South Carolina, Colorado, California.

A *primary* source of information was reported in 99% (70 of 71) of the studies: among these reported sources, 60% (42 of 70) were from medical/hospital records, 36% (25 of 70) from national or regional registers, and 4% (3 of 70) from other sources, such as community-based surveys; a *secondary* source of information was reported in 90% (64 of 71) of the studies: among these reported sources, 58% (37 of 64) were from medical/hospital records, 16% (10 of 64) from associations of patients, 14% (9 of 64) from drug or supplies prescription registers, 8% (5 of 64) from national or regional registers, and 5% (3 of 64) from death certificates and schools registers; finally, a *tertiary* source of information was reported in 21% (15 of 71) of the studies: among these reported sources, 27% (4 of 15) were from national or regional registers, 27% (4 of 15) from associations of patients, 20% (3 of 15) from death certificates, 20% (3 of 15) from drug or supplies prescription registers, and 7% (1 of 15) from medical registers; see details in Table 1. Percentage of ascertainment (completeness) between sources of information was evaluated in 53 of 71 (75%) studies. The mean percentage of ascertainment of these 53 studies was 94% (Table 1).

In the group of young adults (15–19), the lowest incidence of T1D was reported in Mauritius, (1.1/100.000 persons/year) [13], and the highest in Estonia (39.9/100.000 persons/year) [19]. In the 70–79 year age group, the lowest incidence was reported in Navarra, Spain (0.8/100.000 persons/year) [57] and the highest in Kronoberg, Sweden (55/100.000 persons /year) [63]. The details of all retrieved incidence by study and age classes are in Additional file 4: Table S1.

### Diagnostic criteria used to define T1D in adults reported in 71 epidemiological studies

Autoantibodies against beta-cell antigens or the C-peptide were included in the T1D diagnostic criteria in 14 studies [15,30-32,34,35,45,46,54,56,57,63,74,81], detection of ICAs was reported in 9 studies [15,30-32,34,45,46,54,63], IAA in 4 studies [30-32,54], IA2 in 5 studies [30-32,56,57], and GAD in 11 studies [30-32,35,45,46,56,57,63,74,81]. The C-peptide was measured in 7 studies. In one paper difference of auto-antibodies by age group (0–19) was explored but no significant differences were detected [74]. The other reported diagnostic criteria for T1D were the need for insulin therapy (reported in 70 of 71 studies), clinical symptoms of diabetes (reported in 56 of 71 studies), low or normal body weight (14 of 71 studies), and ketosis or ketonuria (26 of 71 studies). The details are shown in Table 1.

### Comparison of adult and children T1D incidences

The variations of incidence of T1D in adults with country and age were studied in each area for which we retrieved information on a geographically defined population. This concerned 35 countries.

#### Variation of T1D incidence with age in adults

**Figure 2:**
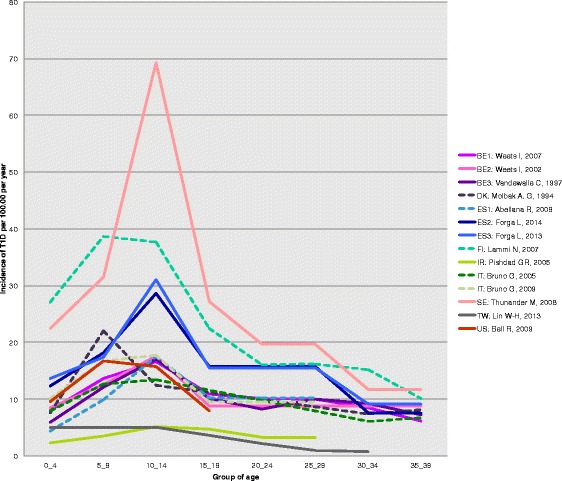
**Age variation of incidence from childhood to adult age.** On this figure, the adult estimates of incidence were taken from the 14 reports of the systematic review using the autoantibodies/C-peptide as diagnostic criteria. Full lines correspond to articles from which both child as well as adult information could be retrieved. The dotted lines are those for which the child information was searched in the same country as in the adult paper, but was from a different paper (see Additional file 3 for details on this literature search). The corresponding countries are shown as: BE1: Belgium (2007) [30]; BE2: Belgium (2002) [31]; BE3: Belgium (1997) [32]; DK: Denmark [34]; ES1: Spain, Catalonia [54]; ES2: Spain, Navarra (2014) [56]; ES3: Spain, Navarra (2013) [57]; FI: Finland [35]; IR: Iran (Islamic Republic of) [15]; IT: Italy [45,46]; SE: Sweden [63], TW: Taiwan [81]; US: United States of America [74].

In 23 out of 35 (66%) countries (55 of 71 studies), the incidence of T1D was higher in the age range of 0–14 compared with 15–19 years. When restricted to the 14 reports for which the criteria of diagnosis of T1D were auto-antibodies against beta-cells or C-peptide detection, the variation of adult incidence with age showed a consistent decrease after the age of 14 years (Figure 2 and Additional file 4: Table S1).

#### Geographical correlation of adult and child T1D incidence

**Figure 3:**
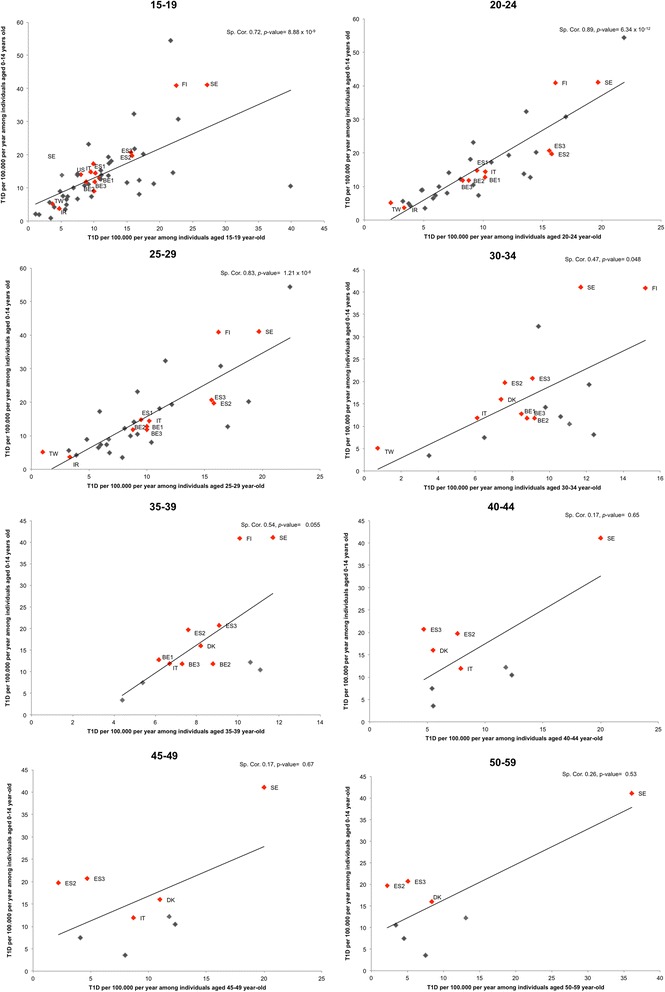
**Geographical correlation of T1D incidence between individuals aged 0–14 years and adults.** Studies using autoantibodies/C-Peptide for T1D case definition are identified by Red diamonds. The corresponding countries are shown as: BE1: Belgium (2007) [30]; BE2: Belgium (2002) [31]; BE3: Belgium (1997) [32]; DK: Denmark [34]; ES1: Spain, Catalonia [54]; ES2: Spain, Navarra (2014) [56]; ES3: Spain, Navarra (2013) [57]; FI: Finland [35]; IR: Iran (Islamic Republic of) [15]; IT: Italy [45,46]; SE: Sweden [63], TW: Taiwan [81]; US: United States of America [74]. Sp. Cor: Spearman correlation.

A significant geographical correlation, as measured by the Spearman correlation coefficient, was found between adult T1D incidence and 0–14 incidence in the age classes 15–19 years, 20–24 years, 25–29 years, 30–34 years and overall in the entire 15–60 group (*r* = 0.75, *p*-value: 5.7 *×* 10^−10^). The correlation was not significant in the oldest class where sparse data were available, but the relation was similar (Figure 3).

### Comparison of male and female T1D adult incidences

T1D incidence was larger in males aged 15 to 39 years than in females in 44 (81%) of the 54 studies reporting incidence by sex (Additional file 5: Table S2). The mean male-to-female ratio in our review was 1.47 (95% CI for mean 1.33-1.60, SD = 0.49, *n* = 54, *p =* < 0.0001).

## Discussion

A first result of this systematic review is the paucity of data available on adult incidence of T1D as compared to those concerning children. The 71 studies retrieved provided information on adult T1D in only 35 countries, 40% of the 88 countries with primary childhood T1D incidence information in the 6th IDF atlas [1].

A second result is that only a small proportion (*n* = 14) of the 71 studies used detection of specific autoantibodies and/or dosage of C-peptide [83] as diagnostic criteria of adult T1D.

A third result was that in a majority of the retrieved studies, adult T1D incidence was greater in men than in women, which contrasts with incidence of T1D in children where sex ratio is around one [2,84]. Using comparative data, Karvonen et al. also described a male excess among young adults in the 15–39 years of age [85]. Sex differences in exposure to possible environmental triggers of T1D, in hormonal/genetic susceptibility, in lifestyle have been proposed as possible explanations for this difference [62].

A last striking observation of the current analysis is the strong geographical correlation of the incidences in adults and children. This correlation may be explained by the fact that adults with T1D share the gene alleles known to be associated to incidence of T1D in children, [86,87], and/or some predisposing environmental causes [4]. For example, in a previous study on incidence of T1D in children, a significant positive correlation was detected between the percentage of urban population and the incidence of T1D in children (*r* = 0.41 *p-*value: < 0.0001) [4]; in this review a significantly higher urban proportion of T1D incidence among adults was found in 4 of the 7 studies reporting differences between rural *vs* urban areas [15,21,42,75].

There was an overall decrease of incidence with age in adults and young adults after the age of 14. A second peak of T1D around the age of 50, as described by Krolewski et al. [88], was only reported in 7% (4 of 58) of the studies [18,63,80,89].

The paucity of data made it impossible to document an increase in adult T1D incidence that would parallel the dramatic increase observed in children [2,3,90]. Indeed, successive studies in the same region over different periods reporting incidence in people aged >30 years of age were only found for Belgium [30-32], Lithuania [20-22] and Sweden [58-62]. Similarly, this review did not dispose of sufficient data to document differences in the clinical presentation of T1D of adults and children as suggested elsewhere [32,40]; indeed only two of the 71 studies describe differences in clinical presentation of T1D between adults and children [89,91].

Improving the quantity and quality of information on adult T1D is not only useful to better understand the epidemiology and natural history of T1D, but can have practical consequences, as delay of T1D diagnosis may mean retardation in insulin treatment, lost opportunities for potential prevention of acute and chronic complications, and even death [92]: in Croatia [18], 14% of the incident cases were identified solely through death certificates, and high mortality was found in the newly-diagnosed T1D aged over 50.

## Conclusions

Overall, the results of this systematic review should encourage the launching of epidemiological studies of adult T1D with specific diagnostic criteria.

### Availability of supporting data

All the supporting data are included as additional files.

## Additional files

Additional file 1:
**Search equation used for the bibliographic analysis.**
Additional file 2:
**PRISMA checklist.**
Additional file 3:
**List of selected papers reporting incidence of T1D in 0–14 year-olds in 9 countries.**
Additional file 4: Table S1. Geographic repartition, and reported adult T1D incidences found in the systematic review. Incidence was per 100.000 persons per year. T1D: Type 1 Diabetes. NW: Nation-wide study. HIGH, LMIC: High, Low-Medium Income Level. Highlighted: reports of the systematic review using the autoantibodies/C-peptide as diagnosis criteria. (a) 0–9 years of age, (b) 10–19 years of age, (c) 10–18 years of age, (d) 15–17 years of age, (e) 15–18 years of age, (−−-): unavailable data. (*): Data was retrieved from a different study; for details see Additional file 3. (†) Studies used in the geographical correlation analyses. (‡) Special population. (§) The five areas were Ohio (8 counties), Washington State (5 counties), South Carolina, Colorado and California; the table presents the mean incidence calculated, retrieved from 5 populations: African American, Asian Pacific Islander, Navajo, Hispanic and non-Hispanic young. Additional file 5: Table S2. T1D incidences by sex in young adults and adults found in the Systematic Review. Male-to-Female ratios >1 are highlighted. Ref: Reference. First author and publication year in reports of the systematic review using the autoantibodies/C-peptide as diagnosis criteria are highlighted. Inc: incidence per 100.000 persons per year. NW: Nation-wide study. HIGH, LMIC: High, Low-Medium Income Level. (†) Studies used for analyses. (§) The five areas were Ohio (8 counties), Washington State (5 counties), South Carolina, Colorado and California; the table presents the mean incidence calculated retrieved from 5 populations: African American, Asian Pacific Islander, Navajo, Hispanic and non-Hispanic young. Incidence was calculated as the mean of retrieved information: (a) in Jews and other non-Arabs and Arabs; (b) in White and Black populations; (c) in Non-Hispanic Whites and Hispanic Whites. (d) Study giving the total incidence by sex, not by age classes.

## References

[1] Patterson C, Guariguata L, Dahlquist G, Soltesz G, Ogle G, Silink M (2013). Diabetes in the young - a global view and worldwide estimates of numbers of children with type 1 diabetes. Diabetes Res Clin Pract.

[2] The DIAMOND Project Group (2006). Incidence and trends of childhood Type 1 diabetes worldwide 1990–1999. The DIAMOND project Group. Diabet Med.

[3] Patterson CC, Gyurus E, Rosenbauer J, Cinek O, Neu A, Schober E (2012). Trends in childhood type 1 diabetes incidence in Europe during 1989–2008: evidence of non-uniformity over time in rates of increase. Diabetologia.

[4] Diaz-Valencia PA, Bougneres P, Valleron AJ (2015). Covariation of the incidence of type 1 diabetes with country characteristics available in public databases. PloS one.

[5] Borg H, Arnqvist HJ, Bjork E, Bolinder J, Eriksson JW, Nystrom L (2003). Evaluation of the new ADA and WHO criteria for classification of diabetes mellitus in young adult people (15–34 yrs) in the Diabetes Incidence Study in Sweden (DISS). Diabetologia.

[6] Tuomi T, Groop LC, Zimmet PZ, Rowley MJ, Knowles W, Mackay IR (1993). Antibodies to glutamic acid decarboxylase reveal latent autoimmune diabetes mellitus in adults with a non-insulin-dependent onset of disease. Diabetes.

[7] Zimmet PZ (1999). Diabetes epidemiology as a tool to trigger diabetes research and care. Diabetologia.

[8] LaPorte RE, Tajima N, Akerblom HK, Berlin N, Brosseau J, Christy M (1985). Geographic differences in the risk of insulin-dependent diabetes mellitus: the importance of registries. Diabetes Care.

[9] Health Statistics and health information systems. [http://www.who.int/healthinfo/global_burden_disease/definition_regions/en/]

[10] LaPorte RE, McCarty D, Bruno G, Tajima N, Baba S (1993). Counting diabetes in the next millennium. Application of capture-recapture technology. Diabetes Care.

[11] GraphClick. In*.*, 3.0 edn: Arizona Software; 2008. Available in the website: http://www.arizona-software.ch/graphclick/ [last accesed: 12 January, 2012].

[12] R Development Core Team: R: A language and environment for statistical computing. R Foundation for Statistical Computing. In*.*, R version 3.0.1 (2013-05-16). http://www.R-project.org/. Vienna, Austria, 2013.

[13] Tuomilehto J, Dabee J, Karvonen M, Dowse GK, Gareeboo H, Virtala E (1993). Incidence of IDDM in Mauritian children and adolescents from 1986 to 1990. Diabetes Care.

[14] Swai AB, Lutale JL, McLarty DG (1993). Prospective study of incidence of juvenile diabetes mellitus over 10 years in Dar es Salaam, Tanzania. BMJ.

[15] Pishdad GR (2005). Low incidence of type 1 diabetes in Iran. Diabetes Care.

[16] Kadiki OA, Reddy MR, Marzouk AA (1996). Incidence of insulin-dependent diabetes (IDDM) and non-insulin-dependent diabetes (NIDDM) (0-34 years at onset) in Benghazi, Libya. Diabetes Res Clin Pract.

[17] Ben Khalifa F, Mekaouar A, Taktak S, Hamhoum M, Jebara H, Kodia A (1997). A five-year study of the incidence of insulin-dependent diabetes mellitus in young Tunisians (preliminary results). Diabetes Metab.

[18] Roglic G, Pavlic-Renar I, Sestan-Crnek S, Prasek M, Kadrnka-Lovrencic M, Radica A (1995). Incidence of IDDM during 1988-1992 in Zagreb, Croatia. Diabetologia.

[19] Kalits I, Podar T (1990). Incidence and prevalence of type 1 (insulin-dependent) diabetes in Estonia in 1988. Diabetologia.

[20] Ostrauskas R, Zalinkevicius R, Jurgeviciene N, Radzeviciene L, Lasaite L (2011). The incidence of type 1 diabetes mellitus among 15-34 years aged Lithuanian population: 18-year incidence study based on prospective databases. BMC Public Health.

[21] Pundziute-Lycka A, Urbonaite B, Ostrauskas R, Zalinkevicius R, Dahlquist GG (2003). Incidence of type 1 diabetes in Lithuanians aged 0-39 years varies by the urban-rural setting, and the time change differs for men and women during 1991-2000. Diabetes Care.

[22] Ostrauskas R, Zalinkevicius R (2000). Incidence in young adulthood-onset Type 1 diabetes mellitus in Lithuania during 1991-1997. Lithuanian Epidemiology Diabetes Study Group. Diabetes Nutr Metab.

[23] Kretowski A, Kowalska I, Peczynska J, Urban M, Green A, Kinalska I (2001). The large increase in incidence of Type I diabetes mellitus in Poland. Diabetologia.

[24] Sobel-Maruniak A, Grzywa M, Orlowska-Florek R, Staniszewski A (2006). The rising incidence of type 1 diabetes in south-eastern Poland. A study of the 0-29 year-old age group, 1980–1999. Endokrynol Pol.

[25] Grzywa MA, Sobel AK (1995). Incidence of IDDM in the province of Rzeszow, Poland, 0- to 29-year-old age-group, 1980-1992. Diabetes Care.

[26] Wysocki MJ, Chanska M, Bak M, Czyzyk AS (1992). Incidence of insulin-dependent diabetes mellitus in Warsaw, Poland, in children and young adults, 1983-1988. World Health Stat Q.

[27] Ionescu-Tirgoviste C, Paterache E, Cheta D, Farcasiu E, Serafinceanu C, Mincu I (1994). Epidemiology of diabetes in Bucharest. Diabet Med.

[28] Kyvik KO, Nystrom L, Gorus F, Songini M, Oestman J, Castell C (2004). The epidemiology of Type 1 diabetes mellitus is not the same in young adults as in children. Diabetologia.

[29] Rami B, Waldhor T, Schober E (2001). Incidence of Type I diabetes mellitus in children and young adults in the province of Upper Austria, 1994-1996. Diabetologia.

[30] Weets I, Rooman R, Coeckelberghs M, De Block C, Van Gaal L, Kaufman JM (2007). The age at diagnosis of type 1 diabetes continues to decrease in Belgian boys but not in girls: a 15-year survey. Diabetes Metab Res Rev.

[31] Weets I, De Leeuw IH, Du Caju MV, Rooman R, Keymeulen B, Mathieu C (2002). The incidence of type 1 diabetes in the age group 0-39 years has not increased in Antwerp (Belgium) between 1989 and 2000: evidence for earlier disease manifestation. Diabetes Care.

[32] Vandewalle CL, Coeckelberghs MI, De Leeuw IH, Du Caju MV, Schuit FC, Pipeleers DG (1997). Epidemiology, clinical aspects, and biology of IDDM patients under age 40 years. Comparison of data from Antwerp with complete ascertainment with data from Belgium with 40% ascertainment. The Belgian Diabetes Registry. Diabetes Care.

[33] Radosevic B, Bukara-Radujkovic G, Miljkovic V, Pejicic S, Bratina N, Battelino T (2013). The incidence of type 1 diabetes in Republic of Srpska (Bosnia and Herzegovina) and Slovenia in the period 1998-2010. Pediatr Diabetes.

[34] Molbak AG, Christau B, Marner B, Borch-Johnsen K, Nerup J (1994). Incidence of insulin-dependent diabetes mellitus in age groups over 30 years in Denmark. Diabet Med.

[35] Lammi N, Taskinen O, Moltchanova E, Notkola IL, Eriksson JG, Tuomilehto J (2007). A high incidence of type 1 diabetes and an alarming increase in the incidence of type 2 diabetes among young adults in Finland between 1992 and 1996. Diabetologia.

[36] Charkaluk ML, Czernichow P, Levy-Marchal C (2002). Incidence data of childhood-onset type I diabetes in France during 1988-1997: the case for a shift toward younger age at onset. Pediatr Res.

[37] Levy-Marchal C (1998). Evolution of the incidence of IDDM in childhood in France. Rev Epidemiol Sante Publique.

[38] Blumenfeld O, Dichtiar R, Shohat T, Israel IRSG (2014). Trends in the incidence of type 1 diabetes among Jews and Arabs in Israel. Pediatr Diabetes.

[39] Sella T, Shoshan A, Goren I, Shalev V, Blumenfeld O, Laron Z (2011). A retrospective study of the incidence of diagnosed Type 1 diabetes among children and adolescents in a large health organization in Israel, 2000-2008. Diabet Med.

[40] Koton S (2007). Incidence of type 1 diabetes mellitus in the 0- to 17-yr-old Israel population, 1997-2003. Pediatr Diabetes.

[41] Garancini P, Gallus G, Calori G, Formigaro F, Micossi P (1991). Incidence and prevalence rates of diabetes mellitus in Italy from routine data: a methodological assessment. Eur J Epidemiol.

[42] Tenconi MT, Devoti G, Albani I, Lorini R, Martinetti M, Fratino P (1995). IDDM in the province of Pavia, Italy, from a population-based registry. A descriptive study. Diabetes Care.

[43] Muntoni S, Songini M (1992). High incidence rate of IDDM in Sardinia. Sardinian Collaborative Group for Epidemiology of IDDM. Diabetes Care.

[44] Frongia O, Mastinu F, Sechi GM (1997). Prevalence and 4-year incidence of insulin-dependent diabetes mellitus in the province of Oristano (Sardinia, Italy). Acta Diabetol.

[45] Bruno G, Novelli G, Panero F, Perotto M, Monasterolo F, Bona G (2009). The incidence of type 1 diabetes is increasing in both children and young adults in Northern Italy: 1984–2004 temporal trends. Diabetologia.

[46] Bruno G, Runzo C, Cavallo-Perin P, Merletti F, Rivetti M, Pinach S (2005). Incidence of type 1 and type 2 diabetes in adults aged 30-49 years: the population-based registry in the province of Turin, Italy. Diabetes Care.

[47] Bruno G, Merletti F, Vuolo A, Pisu E, Giorio M, Pagano G (1993). Sex differences in incidence of IDDM in age-group 15-29 yr. Higher risk in males in Province of Turin, Italy. Diabetes Care.

[48] de Beaufort CE, Michel G, Glaesener G (1988). The incidence of type 1 (insulin-dependent) diabetes mellitus in subjects aged 0-19 years in Luxembourg: a retrospective study from 1977 to 1986. Diabetologia.

[49] Schranz AG, Prikatsky V (1989). Type 1 diabetes in the Maltese Islands. Diabet Med.

[50] Ruwaard D, Hirasing RA, Reeser HM, van Buuren S, Bakker K, Heine RJ (1994). Increasing incidence of type I diabetes in The Netherlands. The second nationwide study among children under 20 years of age. Diabetes Care.

[51] Joner G, Sovik O (1991). The incidence of type 1 (insulin-dependent) diabetes mellitus 15-29 years in Norway 1978-1982. Diabetologia.

[52] Morales-Perez FM, Barquero-Romero J, Perez-Miranda M (2000). Incidence of type I diabetes among children and young adults (0-29 years) in the province of Badajoz, Spain during 1992 to 1996. Acta Paediatr.

[53] Carrillo Dominguez A (2000). Incidence of type 1 diabetes mellitus in the Canary Islands (1995-1996). Epidemiologic Group of the Canary Society of Endocrinology and Nutrition. Rev Clin Esp.

[54] Abellana R, Ascaso C, Carrasco JL, Castell C, Tresserras R (2009). Geographical variability of the incidence of Type 1 diabetes in subjects younger than 30 years in Catalonia, Spain. Med Clin (Barc).

[55] Goday A, Castell C, Tresserras R, Canela J, Taberner JL, Lloveras G (1992). Incidence of type 1 (insulin-dependent) diabetes mellitus in Catalonia, Spain. The Catalan Epidemiology Diabetes Study Group. Diabetologia.

[56] Forga L, Goni MJ, Ibanez B, Cambra K, Mozas D, Chueca M (2014). Incidence of type 1 diabetes in Navarre, 2009-2012. An Sist Sanit Navar.

[57] Forga L, Goni MJ, Cambra K, Ibanez B, Mozas D, Chueca M (2013). En Representacion del Grupo de Estudio de Diabetes tipo 1 de N: [Differences by age and gender in the incidence of type 1 diabetes in Navarre, Spain (2009-2011)]. Gac Sanit/SESPAS.

[58] Dahlquist GG, Nystrom L, Patterson CC (2011). Incidence of type 1 diabetes in Sweden among individuals aged 0-34 years, 1983-2007: an analysis of time trends. Diabetes Care.

[59] Ostman J, Lonnberg G, Arnqvist HJ, Blohme G, Bolinder J, Ekbom Schnell A (2008). Gender differences and temporal variation in the incidence of type 1 diabetes: results of 8012 cases in the nationwide Diabetes Incidence Study in Sweden 1983-2002. J Intern Med.

[60] Pundziute-Lycka A, Dahlquist G, Nystrom L, Arnqvist H, Bjork E, Blohme G (2002). The incidence of Type I diabetes has not increased but shifted to a younger age at diagnosis in the 0-34 years group in Sweden 1983-1998. Diabetologia.

[61] Nystrom L, Dahlquist G, Ostman J, Wall S, Arnqvist H, Blohme G (1992). Risk of developing insulin-dependent diabetes mellitus (IDDM) before 35 years of age: indications of climatological determinants for age at onset. Int J Epidemiol.

[62] Blohme G, Nystrom L, Arnqvist HJ, Lithner F, Littorin B, Olsson PO (1992). Male predominance of type 1 (insulin-dependent) diabetes mellitus in young adults: results from a 5-year prospective nationwide study of the 15-34-year age group in Sweden. Diabetologia.

[63] Thunander M, Petersson C, Jonzon K, Fornander J, Ossiansson B, Torn C (2008). Incidence of type 1 and type 2 diabetes in adults and children in Kronoberg, Sweden. Diabetes Res Clin Pract.

[64] Imkampe AK, Gulliford MC (2011). Trends in Type 1 diabetes incidence in the UK in 0- to 14-year-olds and in 15- to 34-year-olds, 1991-2008. Diabet Med.

[65] Bingley PJ, Gale EA (1989). Incidence of insulin dependent diabetes in England: a study in the Oxford region, 1985-6. BMJ.

[66] Jordan OW, Lipton RB, Stupnicka E, Cruickshank JK, Fraser HS (1994). Incidence of type I diabetes in people under 30 years of age in Barbados, West Indies, 1982-1991. Diabetes Care.

[67] Legault L, Polychronakos C (2006). Annual incidence of type 1 diabetes in Quebec between 1989-2000 in children. Clin Invest Med.

[68] Wagenknecht LE, Roseman JM, Herman WH (1991). Increased incidence of insulin-dependent diabetes mellitus following an epidemic of Coxsackievirus B5. Am J Epidemiol.

[69] Wagenknecht LE, Roseman JM, Alexander WJ (1989). Epidemiology of IDDM in black and white children in Jefferson County, Alabama, 1979-1985. Diabetes.

[70] Vehik K, Hamman RF, Lezotte D, Norris JM, Klingensmith G, Bloch C (2007). Increasing Incidence of Type 1 Diabetes in 0- to 17-Year-Old Colorado Youth. Diabetes Care.

[71] Kostraba JN, Gay EC, Cai Y, Cruickshanks KJ, Rewers MJ, Klingensmith GJ (1992). Incidence of insulin-dependent diabetes mellitus in Colorado. Epidemiology.

[72] Libman IM, LaPorte RE, Becker D, Dorman JS, Drash AL, Kuller L (1998). Was there an epidemic of diabetes in nonwhite adolescents in Allegheny County, Pennsylvania?. Diabetes Care.

[73] Fishbein HA, Faich GA, Ellis SE (1982). Incidence and hospitalization patterns of insulin-dependent diabetes mellitus. Diabetes Care.

[74] Bell RA, Mayer-Davis EJ, Beyer JW, D'Agostino RB, Lawrence JM, Linder B (2009). Diabetes in non-Hispanic white youth: prevalence, incidence, and clinical characteristics: the SEARCH for Diabetes in Youth Study. Diabetes Care.

[75] Allen C, Palta M, D'Alessio DJ (1986). Incidence and differences in urban-rural seasonal variation of type 1 (insulin-dependent) diabetes in Wisconsin. Diabetologia.

[76] Gorham ED, Garland FC, Barrett-Connor E, Garland CF, Wingard DL, Pugh WM (1993). Incidence of insulin-dependent diabetes mellitus in young adults: experience of 1,587,630 US Navy enlisted personnel. Am J Epidemiol.

[77] Tran F, Stone M, Huang CY, Lloyd M, Woodhead HJ, Elliott KD (2014). Population-based incidence of diabetes in Australian youth aged 10-18 yr: increase in type 1 diabetes but not type 2 diabetes. Pediatr Diabetes.

[78] Sutton DL, Lyle DM, Pierce JP (1989). Incidence and prevalence of insulin-dependent diabetes mellitus in the zero- to 19-years' age-group in Sydney. Med J Aust.

[79] Sasaki A, Okamoto N (1992). Epidemiology of childhood diabetes in Osaka District, Japan, using the documents from the medical benefits system specific for childhood diabetes. Diabetes Res Clin Pract.

[80] Scott RS, Brown LJ (1991). Prevalence and incidence of insulin-treated diabetes mellitus in adults in Canterbury, New Zealand. Diabet Med.

[81] Lin WH, Wang MC, Wang WM, Yang DC, Lam CF, Roan JN (2014). Incidence of and mortality from Type I diabetes in Taiwan from 1999 through 2010: a nationwide cohort study. PloS one.

[82] Washington RE, Orchard TJ, Arena VC, Laporte RE, Tull ES (2013). Incidence of type 1 and type 2 diabetes in youth in the U.S. Virgin Islands, 2001-2010. Pediatr Diabetes.

[83] Bingley PJ, Bonifacio E, Ziegler AG, Schatz DA, Atkinson MA, Eisenbarth GS (2001). Proposed guidelines on screening for risk of type 1 diabetes. Diabetes Care.

[84] Soltesz G, Patterson CC, Dahlquist G (2007). Worldwide childhood type 1 diabetes incidence–what can we learn from epidemiology?. Pediatr Diabetes.

[85] Karvonen M, Pitkaniemi M, Pitkaniemi J, Kohtamaki K, Tajima N, Tuomilehto J (1997). Sex difference in the incidence of insulin-dependent diabetes mellitus: an analysis of the recent epidemiological data. World Health Organization DIAMOND Project Group. Diabetes Metab Rev.

[86] Todd JA (2010). Etiology of type 1 diabetes. Immunity.

[87] Caillat-Zucman S, Garchon HJ, Timsit J, Assan R, Boitard C, Djilali-Saiah I (1992). Age-dependent HLA genetic heterogeneity of type 1 insulin-dependent diabetes mellitus. J Clin Invest.

[88] Krolewski AS, Warram JH, Rand LI, Kahn CR (1987). Epidemiologic approach to the etiology of type I diabetes mellitus and its complications. N Engl J Med.

[89] Karjalainen J, Salmela P, Ilonen J, Surcel HM, Knip M (1989). A comparison of childhood and adult type I diabetes mellitus. N Engl J Med.

[90] Patterson CC, Dahlquist GG, Gyurus E, Green A, Soltesz G (2009). Incidence trends for childhood type 1 diabetes in Europe during 1989-2003 and predicted new cases 2005-20: a multicentre prospective registration study. Lancet.

[91] Sabbah E, Savola K, Ebeling T, Kulmala P, Vahasalo P, Ilonen J (2000). Genetic, autoimmune, and clinical characteristics of childhood- and adult-onset type 1 diabetes. Diabetes Care.

[92] Atkinson MA, Eisenbarth GS (2001). Type 1 diabetes: new perspectives on disease pathogenesis and treatment. Lancet.

