# Supplementary material for: Global epidemiology of type 1 diabetes in young adults and adults: a systematic review
Source: BMC Public Health. 2015 Mar 17;15:255. doi: 10.1186/s12889-015-1591-y (PMC4381393; doi:10.1186/s12889-015-1591-y)
Supplement: Additional file 1: — Search equation used for the bibliographic analysis. [file 12889_2015_1591_MOESM1_ESM.docx]

### Additional file 1 – Search equation used for the bibliographic analysis

We used the following equation to search the electronic database Medline on November 6^th^ 2014:

"diabetes mellitus, type 1/epidemiology"[MAJR] AND (("incidence"[MeSH Major Topic] OR incidence[Title/Abstract]) AND (adult[TIAB] OR adult[MeSH Terms]) AND "humans"[MeSH Terms]) AND (Journal Article[PT] NOT ("Diabetic Neuropathies"[MeSH Terms] OR "Diabetic Nephropathies"[MeSH Terms] OR "Cholesterol"[MeSH Terms] OR "Antigens"[MeSH Terms] OR "histocompatibility antigens class ii"[MeSH Terms] OR "insulin"[MeSH Terms] OR "Vitamin D"[MeSH Terms] OR "Diabetic ketoacidosis"[MeSH Terms] OR "Vascular Diseases" [MeSH Terms] OR "Arteriosclerosis" [MeSH Terms] OR "Lipoproteins" [MeSH Terms] OR "Vascular Endothelial Growth Factors" [MeSH Terms] OR "Comorbidity" [MeSH Terms]) 🡺 AND **English**[Lang]: 178 Retrieved articles.

*Note 1. The last terms of the equation (after “Diabetic Neuropathies” were added to focus the bibliographic search on incidence papers by removing all those dealing, for example, with the incidence of complications of T1D.*

*Note 2: Through a general search, we searched in other languages than English. We identified 115 articles published in French and 70 in Spanish, from which we assessed full text for eligibility of 7 papers published in French and 14 in Spanish. At the end, one paper in French and two in Spanish were included in the analysis.*

_______

### *Glossary for PubMed search*: [MAJR]: MeSH Major Topic, a MeSH term that is one of the main topics discussed in the article; [MeSH]: MeSH Terms, the National Library of Medicine's controlled vocabulary of biomedical terms that is used to describe the subject of each indexed journal article in MEDLINE; [SH]: MeSH Subheadings; it describes more completely a particular aspect of a subject; [TIAB]: Title/Abstract, the search include words and numbers included in the title, abstract, and other abstract of a citation; [PT]: Publication Type, it describes the type of material the article represents (e.g., Review, Clinical Trial, Retracted Publication, Letter).
