# Supplementary material for: Global epidemiology of type 1 diabetes in young adults and adults: a systematic review
Source: BMC Public Health. 2015 Mar 17;15:255. doi: 10.1186/s12889-015-1591-y (PMC4381393; doi:10.1186/s12889-015-1591-y)
Supplement: Additional file 3: — List of selected papers reporting incidence of T1D in 0–14 year-olds in 9 countries. [file 12889_2015_1591_MOESM3_ESM.docx]

### Additional file 3 – List of selected papers reporting incidence of T1D in 0-14 years-olds among 9 countries.

This list was obtained through a separate review focused on the 9 countries considered (1st column). These values were used in the child-adult correlations.

| **Country, Area reported in the article** | **First Author, publication year** | **Mean TID incidence group of age 0-14 years** | **Period of study** | Ref. |
| --- | --- | --- | --- | --- |
| Australia: New South Wales | Diamond, 2006 | **14.5** | 1990-1993 | [1] |
| Slovakia: NW | Diamond, 2006 | **8.90** | 1990-1999 | [1] |
| Denmark: Copenhagen and Frederiksborg | Eurodiab, 2000 | **16.00** | 1989-1994 | [2] |
| Finland: NW | Diamond, 2006 | **40.90** | 1990-1999 | [1] |
| Italy: Turin | Bruno G, 2009 | **11.86** | 1984-2004 | [3] |
| Italy: Turin | Bruno G, 2009 | **14.8** | 2000-2004 | [3] |
| Norway: NW | Joner G, 1989 | **20.22** | 1973-1982 | [4] |
| Lithuania: NW | Patterson CC, 2012 | **14.20** | 2004-2008 | [5] |
| United States: Wisconsin | Allen C, 1986 | **18.10** | 1970-1979 | [6] |

NW: Nation-wide

**References**

1. The DIAMOND Project Group: **Incidence and trends of childhood Type 1 diabetes worldwide 1990-1999. The DIAMOND project Group**. *Diabet Med* 2006, **23**(8):857-866.

2. EURODIAB ACE Study Group: **Variation and trends in incidence of childhood diabetes in Europe**. *Lancet* 2000, **355**(9207):873-876.

3. Bruno G, Novelli G, Panero F, Perotto M, Monasterolo F, Bona G, Perino A, Rabbone I, Cavallo-Perin P, Cerutti F: **The incidence of type 1 diabetes is increasing in both children and young adults in Northern Italy: 1984–2004 temporal trends**. *Diabetologia* 2009, **52**(12):2531-2535.

4. Joner G, Sovik O: **Increasing incidence of diabetes mellitus in Norwegian children 0-14 years of age 1973-1982**. *Diabetologia* 1989, **32**(2):79-83.

5. Patterson CC, Gyurus E, Rosenbauer J, Cinek O, Neu A, Schober E, Parslow RC, Joner G, Svensson J, Castell C *et al*: **Trends in childhood type 1 diabetes incidence in Europe during 1989-2008: evidence of non-uniformity over time in rates of increase**. *Diabetologia* 2012, **55**(8):2142-2147.

6. Allen C, Palta M, D'Alessio DJ: **Incidence and differences in urban-rural seasonal variation of type 1 (insulin-dependent) diabetes in Wisconsin**. *Diabetologia* 1986, **29**(9):629-633.
