# Supplementary material for: Global epidemiology of type 1 diabetes in young adults and adults: a systematic review
Source: BMC Public Health. 2015 Mar 17;15:255. doi: 10.1186/s12889-015-1591-y (PMC4381393; doi:10.1186/s12889-015-1591-y)
Supplement: Additional file 4: Table S1. — Geographic repartition, and reported adult T1D incidences found in the systematic review. Incidence was per 100.000 persons per year. T1D: Type 1 Diabetes. NW: Nation-wide study. HIGH, LMIC: High, Low-Medium Income Level. Highlighted: reports of the systematic review using the autoantibodies/C-peptide as diagnosis criteria. (a) 0–9 years of age, (b) 10–19 years of age, (c) 10–18 years of age, (d) 15–17 years of age, (e) 15–18 years of age, (−−-): unavailable data. (*): Data was retrieved from a different study; for details see Additional file 3. (†) Studies used in the geographical correlation analyses. (‡) Special population. (§) The five areas were Ohio (8 counties), Washington State (5 counties), South Carolina, Colorado and California; the table presents the mean incidence calculated, retrieved from 5 populations: African American, Asian Pacific Islander, Navajo, Hispanic and non-Hispanic young. [file 12889_2015_1591_MOESM4_ESM.docx]

## Additional file 4: Table S1 - Geographic repartition, and reported adult T1D incidences found in the systematic review

| **Study information** | | | | | **Mean T1D incidence by age group** | | | | | | | | | | | |
| --- | --- | --- | --- | --- | --- | --- | --- | --- | --- | --- | --- | --- | --- | --- | --- | --- |
| **Country, Area** | **First Author, publication year** | **Ref** | **Age range** | **Period** | **0-14** | **15-19** | **20-24** | **25-29** | **30-34** | **35-39** | **40-44** | **45-49** | **50-59** | **60-69** | **70-79** | **80-100** |
| **African Region, LMIC** |  |  |  |  |  |  |  |  |  |  |  |  |  |  |  |  |
| Mauritius: NW | Tuomilehto J., 1993 † | [1] | 0-19 | 1986-1990 | 2.1 | 1.1 | --- | --- | --- | --- | --- | --- | --- | --- | --- | --- |
| United Republic of Tanzania:  Dar es Salaam | Swai A. B., 1993 † | [2] | 0-19 | 1982-1991 | 0.9 | 3.4 | --- | --- | --- | --- | --- | --- | --- | --- | --- | --- |
| **Eastern Mediterranean Region, LMIC** | |  |  |  |  |  |  |  |  |  |  |  |  |  |  |  |
| Iran (Islamic Republic of): Fars | **Pishdad G. R., 2005 †** | [3] | 0-29 | 1990-1994 | 3.7 | 4.7 | 3.4 | 3.3 | --- | --- | --- | --- | --- | --- | --- | --- |
| Libyan Arab Jamahiriya:  Benghazi | Kadiki O. A., 1996 † | [4] | 0-34 | 1981-1990 | 8.1 | 16.9 | 7.0 | 10.4 | 12.4 | --- | --- | --- | --- | --- | --- | --- |
| Tunisia: Beja, Monastir, Gafsa | Ben Khalifa F., 1998 † | [5] | 0-19 | 1990-1994 | 6.7 | 7.5 | --- | --- | --- | --- | --- | --- | --- | --- | --- | --- |
| **European Region, LMIC** |  |  |  |  |  |  |  |  |  |  |  |  |  |  |  |  |
| Croatia: Zagreb | Roglic G., 1995 † | [6] | 0->55 | 1988-1992 | 7.4 | 9.6 | 9.6 | 6.5 | 6.5 | 5.4 | 5.4 | 4.1 | 4.5 | --- | --- | --- |
| Estonia: NW | Kalits I., 1990 † | [7] | 0->50 | 1988-1988 | 10.5 | 39.9 | 9.2 | 9.2 | 11.1 | 11.1 | 12.3 | 12.3 | 3.4 | --- | --- | --- |
| Lithuania: NW | Ostrauskas R., 2011 † | [8] | 15-34 | 1991-2008 | 14.2* | 7.4 | 7.1 | 8.9 | 9.8 |  | --- | --- | --- | --- | --- | --- |
| Lithuania: NW | Pundziute-Lycka A., 2003 | [9] | 0-39 | 1991-2000 | 8.4^(a)^ | 11.1^(b)^ | 7.8 | 7.8 | 7.8 | 7.8 | --- | --- | --- | --- | --- | --- |
| Lithuania: NW | Ostrauskas R., 2000 | [10] | 15-39 | 1991-1997 | --- | 6.9 | 6.9 | 5.5 | 8.8 | 7.8 | --- | --- | --- | --- | --- | --- |
| Poland: Bialystok | Kretowski A., 2001 † | [11] | 0-29 | 1994-1998 | 7.3 | 6.0 | 6.0 | 6.0 | --- | --- | --- | --- | --- | --- | --- | --- |
| Poland: Province of Rzeszow | Sobel-Maruniak A., 2006 † | [12] | 0-29 | 1980-1999 | 6.4 | 5.8 | 5.8 | 5.8 | --- | --- | --- | --- | --- | --- | --- | --- |
| Poland: Province of Rzeszow | Grzywa M. A., 1995 | [13] | 0-29 | 1980-1992 | 5.4 | 6.0 | 5.0 | 5.7 | --- | --- | --- | --- | --- | --- | --- | --- |
| Poland: Warsaw | Wysocki M. J., 1992 † | [14] | 0-29 | 1983-1988 | 4.87 | 5.25 | 3.75 | 6.75 | --- | --- | --- | --- | --- | --- | --- | --- |
| Romania: Bucharest | Ionescu-Tirgoviste C., 1994 † | [15] | 0-≥85 | 1981-1991 | 3.5 | 5.6 | 5.1 | 7.9 | 3.5 | 4.4 | 5.5 | 8.0 | 7.5 | 9.2 | 8.6 | 3.1 |
| Slovakia: NW | Kyvik K. O., 2004 † | [16] | 15-29 | 1996-1997 | 8.9* | 4.8 | 4.8 | 4.8 | --- | --- | --- | --- | --- | --- | --- | --- |
| **European Region, HIGH** |  |  |  |  |  |  |  |  |  |  |  |  |  |  |  |  |
| Austria: Upper | Rami B., 2001 † | [17] | 0-29 | 1994-1996 | 9.0 | 10.0 | 4.9 | 6.7 | --- | --- | --- | --- | --- | --- | --- | --- |
| Belgium: Antwerp | **Weets I., 2007 †** | [18] | 0-39 | 1989-2003 | 12.8 | 10.9 | 10.2 | 10.0 | 8.5 | 6.2 | --- | --- | --- | --- | --- | --- |
| Belgium: Antwerp | **Weets I., 2002 †** | [19] | 0-39 | 1989-2000 | 11.8 | 8.8 | 8.8 | 8.8 | 8.8 | 8.8 |  |  |  |  |  |  |
| Belgium: Antwerp | **Vandewalle C., 1997 †** | [20] | 0-39 | 1989-1995 | 11.8 | 10.1 | 8.3 | 10.0 | 9.20 | 7.3 | --- | --- | --- | --- | --- | --- |
| Bosnia and Herzegovina:  Republic of Srpska | Radosevic B., 2013 † | [21] | 0-18 | 1998-2010 | 13.8 | 5.1 ^(e)^ | --- | --- | --- | --- | --- | --- | --- | --- | --- | --- |
| Denmark: Copenhagen and  Frederiksborg | **Molbak A. G., 1994 †** | [22] | 30-95 | 1973-1977 | 16.0* | --- | --- | --- | 7.4 | 8.2 | 5.5 | 11.0 | 8.4 | 10.4 | 11.2 | 4.9 |
| Finland: NW | **Lammi N., 2007 †** | [23] | 15-39 | 1992-1996 | 40.9* | 22.5 | 16.1 | 16.2 | 15.2 | 10.1 | --- | --- | --- | --- | --- | --- |
| France: Aquitaine, Lorraine,  Basse Normandie, Haute  Normandie | Charkaluk M. L., 2002 † | [24] | 0-19 | 1988-1997 | 8.9 | 6.1 | --- | --- | --- | --- | --- | --- | --- | --- | --- | --- |
| France: Aquitaine, Lorraine,  Basse Normandie, Haute  Normandie | Levy-Marchal C., 1998 | [25] | 0-19 | 1988-1995 | 7.6 | 5.3 | --- | --- | --- | --- | --- | --- | --- | --- | --- | --- |
| Israel: NW | Blumenfeld O., 2014 † | [26] | 0-17 | 1997-2010 | 11.1 | 9.2^(d)^ | --- | --- | --- | --- | --- | --- | --- | --- | --- | --- |
| Israel: NW | Sella T., 2011 | [27] | 0-17 | 2000-2008 | 13.3 | 11.1^(d)^ | --- | --- | --- | --- | --- | --- | --- | --- | --- | --- |
| Israel: NW | Koton S., 2007 | [28] | 0-17 | 1997-2003 | 9.2 | 8.8 ^(d)^ | --- | --- | --- | --- | --- | --- | --- | --- | --- | --- |
| Italy: Lombardie | Garancini P., 1991 † | [29] | 0-34 | 1981-1982 | 4.2 | 3.8 | 3.7 | 3.0 | 5.0 | --- | --- | --- | --- | --- | --- | --- |
| Italy: Pavia | Tenconi M. T., 1995 † | [30] | 0-29 | 1988-1992 | 10.0 | 6.9 | 6.3 | 8.6 | --- | --- | --- | --- | --- | --- | --- | --- |
| Italy: Sardinia (Oristano) | Frongia O., 1997 **†** | [31] | 0-29 | 1993-1996 | 54.4 | 21.6 | 21.9 | 22.4 | --- | --- | --- | --- | --- | --- | --- | --- |
| Italy: Sardinia | Muntoni S., 1992 † | [32] | 0-29 | 1989-1990 | 30.7 | 22.8 | 17.0 | 16.4 | --- | --- | --- | --- | --- | --- | --- | --- |
| Italy: Turin | **Bruno G., 2009 †** | [33] | 15-29 | 2000-2004 | 14.8* | 9.5 | 9.5 | 9.5 | --- | --- | --- | --- | --- | --- | --- | --- |
| Italy: Turin | **Bruno G., 2005 †** | [34] | 30-49 | 1999-2001 | 11.9* | --- | --- | --- | 6.1 | 6.7 | 7.9 | 8.7 | --- | --- | --- | --- |
| Italy: Turin | Bruno G., 1993 | [35] | 0-29 | 1984-1988 | 8.2 | 6.9 | 5.9 | 4.6 | --- | --- | --- | --- | --- | --- | --- | --- |
| Luxembourg: NW | De Beaufort C. E., 1988 † | [36] | 0-19 | 1977-1986 | 10.7 | 8.5 | --- | --- | --- | --- | --- | --- | --- | --- | --- | --- |
| Malta: NW | Schranz A. G., 1989 † | [37] | 0-24 | 1980-1987 | 13.8 | 12.2 | 13.5 | --- | --- | --- | --- | --- | --- | --- | --- | --- |
| Netherlands: NW | Ruwaard D., 1994 † | [38] | 0-19 | 1988-1990 | 11.5 | 15.0 | --- | --- | --- | --- | --- | --- | --- | --- | --- | --- |
| Norway: NW | Joner G., 1991 † | [39] | 15-29 | 1978-1982 | 20.2* | 17.5 | 14.5 | 18.8 | --- | --- | --- | --- | --- | --- | --- | --- |
| Slovenia: NW | Radosevic B., 2013 † | [21] | 0-18 | 1998-2010 | 13.8 | 7.5 ^(e)^ | --- | --- | --- | --- | --- | --- | --- | --- | --- | --- |
| Spain: Badajoz | Morales-Perez F. M., 2000 † | [40] | 0-29 | 1992-1996 | 17.2 | 9.9 | 10.7 | 5.9 | --- | --- | --- | --- | --- | --- | --- | --- |
| Spain: Canarias Islands | Carrillo Dominguez A., 2000 † | [41] | 0-30 | 1995-1996 | 23.2 | 9.2 | 9.2 | 9.2 | --- | --- | --- | --- | --- | --- | --- | --- |
| Spain: Catalonia | **Abellana R., 2009 †** | [42] | 0-29 | 1989-1998 | 14.4 | 10.2 | 10.2 | 10.2 | --- | --- | --- | --- | --- | --- | --- | --- |
| Spain: Catalonia | Goday A. 1992 | [43] | 0-29 | 1987-1990 | 11.5 | 11.4 | 11.3 | 8.5 | --- | --- | --- | --- | --- | --- | --- | --- |
| Spain: Navarra | **Forga L., 2014 †** | [44] | 0->45 | 2009-2012 | 19.7 | 15.8 | 15.8 | 15.8 | 7.6 | 7.6 | 7.6 | 2.2 | 2.2 | 2.2 | 2.2 | --- |
| Spain: Navarra | **Forga L., 2013 †** | [45] | 0-79 | 2009-2011 | 20.7 | 15.6 | 15.6 | 15.6 | 9.1 | 9.1 | 4.7 | 4.7 | 5.1 | 0.5 | 0.7 | --- |
| Sweden: NW | Dahlquist G. G., 2011 † | [46] | 0-34 | 1983-2007 | 32.3 | 16.1 | 13.6 | 11.6 | 9.4 | --- | --- | --- | --- | --- | --- | --- |
| Sweden: NW | Östman J., 2008 | [47] | 15-34 | 1983-2002 | --- | 14.8 | 13.9 | 12.5 | 9.8 | --- | --- | --- | --- | --- | --- | --- |
| Sweden: NW | Pundziute-Lycka A., 2002 | [48] | 0-34 | 1983-1998 | 28.0 | 15.2 | 13.2 | 12.1 | 9.9 | --- | --- | --- | --- | --- | --- | --- |
| Sweden: NW | Nyström L., 1992 | [49] | 0-34 | 1983-1987 | 25.9 | 14.7 | 12.8 | 11.7 | 9.7 | --- | --- | --- | --- | --- | --- | --- |
| Sweden: NW | Blohme G., 1992 | [50] | 15-34 | 1983-1987 | --- | 14.8 | 12.7 | 11.7 | 9.8 | --- | --- | --- | --- | --- | --- | --- |
| Sweden: Kronoberg | **Thunander M., 2008 †** | [51] | 0-100 | 1998-2001 | 41.1 | 27.2 | 19.7 | 19.7 | 11.7 | 11.7 | 20.0 | 20.0 | 36.1 | 35.3 | 55.0 | 27.3 |
| United Kingdom: NW | Imkampe A. K., 2011 † | [52] | 0-34 | 1991-2008 | 19.3 | 12.2 | 12.2 | 12.2 | 12.2 | --- | --- | --- | --- | --- | --- | --- |
| United Kingdom: Oxford  region | Bingley P. J., 1989 | [53] | 0-21 | 1985-1986 | 15.7 | 16.2 | --- | --- | --- | --- | --- | --- | --- | --- | --- | --- |
| **Region of the Americas, LMIC** | |  |  |  |  |  |  |  |  |  |  |  |  |  |  |  |
| Barbados: NW | Jordan O. W., 1994 † | [54] | 0-29 | 1982-1991 | 5.6 | 3.3 | 3.3 | 3.3 | --- | --- | --- | --- | --- | --- | --- | --- |
| **Region of the Americas, HIGH** | |  |  |  |  |  |  |  |  |  |  |  |  |  |  |  |
| Canada: Quebec | Legault L., 2006 † | [55] | 0-18 | 2000 | 15.3 | 11.1 ^(d)^ | --- | --- | --- | --- | --- | --- | --- | --- | --- | --- |
| United States of America:  Alabama (Jefferson County) | Wagenknecht L. E., 1991 † | [56] | 0-19 | 1979-1988 | 13.9 | 11.1 | --- | --- | --- | --- | --- | --- | --- | --- | --- | --- |
| United States of America:  Alabama (Jefferson County) | Wagenknecht L. E., 1989 | [57] | 0-19 | 1979-1985 | 18.3 | 9.3 | --- | --- | --- | --- | --- | --- | --- | --- | --- | --- |
| United States of America:  Colorado | Vehik K., 2007 † | [58] | 0-17 | 2000-2004 | 21.8 | 16.1 ^(d)^ | --- | --- | --- | --- | --- | --- | --- | --- | --- | --- |
| United States of America:  Colorado | Kostraba J. N., 1992 | [59] | 0-17 | 1978-1988 | 12.8 | 8.7 | --- | --- | --- | --- | --- | --- | --- | --- | --- | --- |
| United States of America:  Pennsylvania (Allegheny) | Libman I. M., 1998 † | [60] | 0-19 | 1990-1994 | 17.4 | 12.3 | --- | --- | --- | --- | --- | --- | --- | --- | --- | --- |
| United States of America:  Rhode Island | Fishbein H. A., 1982 † | [61] | 0-29 | 1979-1980 | 12.7 | 11.0 | 14.0 | 17.0 | --- | --- | --- | --- | --- | --- | --- | --- |
| United States of America: five  areas ^§^ | **Bell R., 2009 †** | [62] | 0-19 | 2002-2005 | 14.0 | 8.0 | --- | --- | --- | --- | --- | --- | --- | --- | --- | --- |
| United States of America:  Wisconsin | Allen C., 1986 † | [63] | 0-29 | 1970-1979 | 18.1 | 12.7 | 9.0 | 11.1 | --- | --- | --- | --- | --- | --- | --- | --- |
| United States of America: The  United States Navy | Gorham C., 1993 ‡ | [64] | 17-34 | 1974-1988 | 18.1 | 10.4 | 21.8 | 27.3 | 61.7 | --- | --- | --- | --- | --- | --- | --- |
| **Western Pacific Region, HIGH** | |  |  |  |  |  |  |  |  |  |  |  |  |  |  |  |
| Australia: New South Wales | Tran F., 2014 † | [65] | 10-18 | 2001-2008 | 14.5* | 22.0^(c)^ | --- | --- | --- | --- | --- | --- | --- | --- | --- | --- |
| Australia: Sydney (Southern  Metropolitan Heath Region) | Sutton L., 1989 † | [66] | 0-19 | 1984-1987 | 12.91 | 10.78 | --- | --- | --- | --- | --- | --- | --- | --- | --- | --- |
| Japan: Osaka | Sasaki A., 1992 † | [67] | 0-18 | 1978-1988 | 1.89 | 1.65^(e)^ | --- | --- | --- | --- | --- | --- | --- | --- | --- | --- |
| New Zealand: Canterbury | Scott, R. S., 1991† | [68] | 0-≥80 | 1981-1986 | 12.2 | 16.9 ^(b)^ | 8.1 | 8.1 | 10.6 | 10.6 | 11.8 | 11.8 | 13.1 | 18.6 | 21.7 | --- |
| **Other Regions currently non-WHO** | |  |  |  |  |  |  |  |  |  |  |  |  |  |  |  |
| Taiwan: NW | **Lin W. -H., 2013 †** | [69] | 0-≥60 | 1999-2010 | 5.1 | 3.68 | 2.23 | 0.98 | 0.73 | --- | --- | --- | --- | --- | --- | --- |
| US Virgin Islands: NW | Washington R. E., 2013 † | [70] | 0-19 | 2001-2010 | 11.2^(a)^ | 19.1^(b)^ | --- | --- | --- | --- | --- | --- | --- | --- | --- | --- |

## Incidence was per 100.000 persons per year. T1D: Type 1 Diabetes. NW: Nation-wide study. HIGH, LMIC: High, Low-Medium Income Level. Highlighted: reports of the systematic review using the autoantibodies/C-peptide as diagnosis criteria. (a) 0-9 years of age, (b) 10-19 years of age, (c) 10-18 years of age, (d) 15-17 years of age, (e) 15-18 years of age, (---): unavailable data. (*): Data was retrieved from a different study; for details see Additional file 3. (†) Studies used in the geographical correlation analyses. (‡) Special population. (§) The five areas were Ohio (8 counties), Washington State (5 counties), South Carolina, Colorado and California; the table presents the mean incidence calculated retrieved from 5 populations: African American, Asian Pacific Islander, Navajo, Hispanic and non-Hispanic young.

**References**

1. Tuomilehto J, Dabee J, Karvonen M, Dowse GK, Gareeboo H, Virtala E, Tiihonen M, Alberti KG, Zimmet PZ: **Incidence of IDDM in Mauritian children and adolescents from 1986 to 1990**. *Diabetes care* 1993, **16**(12):1588-1591.

2. Swai AB, Lutale JL, McLarty DG: **Prospective study of incidence of juvenile diabetes mellitus over 10 years in Dar es Salaam, Tanzania**. *BMJ* 1993, **306**(6892):1570-1572.

3. Pishdad GR: **Low incidence of type 1 diabetes in Iran**. *Diabetes care* 2005, **28**(4):927-928.

4. Kadiki OA, Reddy MR, Marzouk AA: **Incidence of insulin-dependent diabetes (IDDM) and non-insulin-dependent diabetes (NIDDM) (0-34 years at onset) in Benghazi, Libya**. *Diabetes research and clinical practice* 1996, **32**(3):165-173.

5. Ben Khalifa F, Mekaouar A, Taktak S, Hamhoum M, Jebara H, Kodia A, Zouari B, Chakroun M: **A five-year study of the incidence of insulin-dependent diabetes mellitus in young Tunisians (preliminary results)**. *Diabetes Metab* 1997, **23**(5):395-401.

6. Roglic G, Pavlic-Renar I, Sestan-Crnek S, Prasek M, Kadrnka-Lovrencic M, Radica A, Metelko Z: **Incidence of IDDM during 1988-1992 in Zagreb, Croatia**. *Diabetologia* 1995, **38**(5):550-554.

7. Kalits I, Podar T: **Incidence and prevalence of type 1 (insulin-dependent) diabetes in Estonia in 1988**. *Diabetologia* 1990, **33**(6):346-349.

8. Ostrauskas R, Zalinkevicius R, Jurgeviciene N, Radzeviciene L, Lasaite L: **The incidence of type 1 diabetes mellitus among 15-34 years aged Lithuanian population: 18-year incidence study based on prospective databases**. *BMC public health* 2011, **11**:813.

9. Pundziute-Lycka A, Urbonaite B, Ostrauskas R, Zalinkevicius R, Dahlquist GG: **Incidence of type 1 diabetes in Lithuanians aged 0-39 years varies by the urban-rural setting, and the time change differs for men and women during 1991-2000**. *Diabetes care* 2003, **26**(3):671-676.

10. Ostrauskas R, Zalinkevicius R: **Incidence in young adulthood-onset Type 1 diabetes mellitus in Lithuania during 1991-1997. Lithuanian Epidemiology Diabetes Study Group**. *Diabetes, nutrition & metabolism* 2000, **13**(2):68-74.

11. Kretowski A, Kowalska I, Peczynska J, Urban M, Green A, Kinalska I: **The large increase in incidence of Type I diabetes mellitus in Poland**. *Diabetologia* 2001, **44 Suppl 3**:B48-50.

12. Sobel-Maruniak A, Grzywa M, Orlowska-Florek R, Staniszewski A: **The rising incidence of type 1 diabetes in south-eastern Poland. A study of the 0-29 year-old age group, 1980-1999**. *Endokrynol Pol* 2006, **57**(2):127-130.

13. Grzywa MA, Sobel AK: **Incidence of IDDM in the province of Rzeszow, Poland, 0- to 29-year-old age-group, 1980-1992**. *Diabetes care* 1995, **18**(4):542-544.

14. Wysocki MJ, Chanska M, Bak M, Czyzyk AS: **Incidence of insulin-dependent diabetes mellitus in Warsaw, Poland, in children and young adults, 1983-1988**. *World Health Stat Q* 1992, **45**(4):315-320.

15. Ionescu-Tirgoviste C, Paterache E, Cheta D, Farcasiu E, Serafinceanu C, Mincu I: **Epidemiology of diabetes in Bucharest**. *Diabetic medicine : a journal of the British Diabetic Association* 1994, **11**(4):413-417.

16. Kyvik KO, Nystrom L, Gorus F, Songini M, Oestman J, Castell C, Green A, Guyrus E, Ionescu-Tirgoviste C, McKinney PA *et al*: **The epidemiology of Type 1 diabetes mellitus is not the same in young adults as in children**. *Diabetologia* 2004, **47**(3):377-384.

17. Rami B, Waldhor T, Schober E: **Incidence of Type I diabetes mellitus in children and young adults in the province of Upper Austria, 1994-1996**. *Diabetologia* 2001, **44 Suppl 3**:B45-47.

18. Weets I, Rooman R, Coeckelberghs M, De Block C, Van Gaal L, Kaufman JM, Keymeulen B, Mathieu C, Weber E, Pipeleers DG *et al*: **The age at diagnosis of type 1 diabetes continues to decrease in Belgian boys but not in girls: a 15-year survey**. *Diabetes Metab Res Rev* 2007, **23**(8):637-643.

19. Weets I, De Leeuw I, Du Caju M, Rooman R, Keymeulen B, Mathieu C, Rottiers R, Daubresse J, Rocour-Brumioul D, Pipeleers D *et al*: **The incidence of type 1 diabetes in the age group 0-39 years has not increased in Antwerp (Belgium) between 1989 and 2000: evidence for earlier disease manifestation**. *Diabetes Care* 2002, **25**:840-846.

20. Vandewalle CL, Coeckelberghs MI, De Leeuw IH, Du Caju MV, Schuit FC, Pipeleers DG, Gorus FK: **Epidemiology, clinical aspects, and biology of IDDM patients under age 40 years. Comparison of data from Antwerp with complete ascertainment with data from Belgium with 40% ascertainment. The Belgian Diabetes Registry**. *Diabetes care* 1997, **20**(10):1556-1561.

21. Radosevic B, Bukara-Radujkovic G, Miljkovic V, Pejicic S, Bratina N, Battelino T: **The incidence of type 1 diabetes in Republic of Srpska (Bosnia and Herzegovina) and Slovenia in the period 1998-2010**. *Pediatric diabetes* 2013, **14**(4):273-279.

22. Molbak AG, Christau B, Marner B, Borch-Johnsen K, Nerup J: **Incidence of insulin-dependent diabetes mellitus in age groups over 30 years in Denmark**. *Diabetic medicine : a journal of the British Diabetic Association* 1994, **11**(7):650-655.

23. Lammi N, Taskinen O, Moltchanova E, Notkola IL, Eriksson JG, Tuomilehto J, Karvonen M: **A high incidence of type 1 diabetes and an alarming increase in the incidence of type 2 diabetes among young adults in Finland between 1992 and 1996.** *Diabetologia* 2007, **50**(7):1393-1400.

24. Charkaluk ML, Czernichow P, Levy-Marchal C: **Incidence data of childhood-onset type I diabetes in France during 1988-1997: the case for a shift toward younger age at onset**. *Pediatr Res* 2002, **52**(6):859-862.

25. Levy-Marchal C: **[Evolution of the incidence of IDDM in childhood in France]**. *Revue d'epidemiologie et de sante publique* 1998, **46**(3):157-163.

26. Blumenfeld O, Dichtiar R, Shohat T, Israel IRSG: **Trends in the incidence of type 1 diabetes among Jews and Arabs in Israel**. *Pediatric diabetes* 2014, **15**(6):422-427.

27. Sella T, Shoshan A, Goren I, Shalev V, Blumenfeld O, Laron Z, Chodick G: **A retrospective study of the incidence of diagnosed Type 1 diabetes among children and adolescents in a large health organization in Israel, 2000-2008**. *Diabetic medicine : a journal of the British Diabetic Association* 2011, **28**(1):48-53.

28. Koton S: **Incidence of type 1 diabetes mellitus in the 0- to 17-yr-old Israel population, 1997-2003**. *Pediatric diabetes* 2007, **8**(2):60-66.

29. Garancini P, Gallus G, Calori G, Formigaro F, Micossi P: **Incidence and prevalence rates of diabetes mellitus in Italy from routine data: a methodological assessment**. *European journal of epidemiology* 1991, **7**(1):55-63.

30. Tenconi MT, Devoti G, Albani I, Lorini R, Martinetti M, Fratino P, Ferrari E, Ferrero E, Severi F: **IDDM in the province of Pavia, Italy, from a population-based registry.A descriptive study**. *Diabetes care* 1995, **18**(7):1017-1019.

31. Frongia O, Mastinu F, Sechi GM: **Prevalence and 4-year incidence of insulin-dependent diabetes mellitus in the province of Oristano (Sardinia, Italy)**. *Acta Diabetol* 1997, **34**(3):199-205.

32. Muntoni S, Songini M: **High incidence rate of IDDM in Sardinia. Sardinian Collaborative Group for Epidemiology of IDDM**. *Diabetes care* 1992, **15**(10):1317-1322.

33. Bruno G, Novelli G, Panero F, Perotto M, Monasterolo F, Bona G, Perino A, Rabbone I, Cavallo-Perin P, Cerutti F: **The incidence of type 1 diabetes is increasing in both children and young adults in Northern Italy: 1984–2004 temporal trends**. *Diabetologia* 2009, **52**(12):2531-2535.

34. Bruno G, Runzo C, Cavallo-Perin P, Merletti F, Rivetti M, Pinach S, Novelli G, Trovati M, Cerutti F, Pagano G: **Incidence of type 1 and type 2 diabetes in adults aged 30-49 years: the population-based registry in the province of Turin, Italy**. *Diabetes care* 2005, **28**(11):2613-2619.

35. Bruno G, Merletti F, Vuolo A, Pisu E, Giorio M, Pagano G: **Sex differences in incidence of IDDM in age-group 15-29 yr. Higher risk in males in Province of Turin, Italy**. *Diabetes care* 1993, **16**(1):133-136.

36. de Beaufort CE, Michel G, Glaesener G: **The incidence of type 1 (insulin-dependent) diabetes mellitus in subjects aged 0-19 years in Luxembourg: a retrospective study from 1977 to 1986**. *Diabetologia* 1988, **31**(10):758-761.

37. Schranz AG, Prikatsky V: **Type 1 diabetes in the Maltese Islands**. *Diabetic medicine : a journal of the British Diabetic Association* 1989, **6**(3):228-231.

38. Ruwaard D, Hirasing RA, Reeser HM, van Buuren S, Bakker K, Heine RJ, Geerdink RA, Bruining GJ, Vaandrager GJ, Verloove-Vanhorick SP: **Increasing incidence of type I diabetes in The Netherlands. The second nationwide study among children under 20 years of age**. *Diabetes care* 1994, **17**(6):599-601.

39. Joner G, Sovik O: **The incidence of type 1 (insulin-dependent) diabetes mellitus 15-29 years in Norway 1978-1982**. *Diabetologia* 1991, **34**(4):271-274.

40. Morales-Perez FM, Barquero-Romero J, Perez-Miranda M: **Incidence of type I diabetes among children and young adults (0-29 years) in the province of Badajoz, Spain during 1992 to 1996**. *Acta Paediatr* 2000, **89**(1):101-104.

41. Carrillo Dominguez A: **[Incidence of type 1 diabetes mellitus in the Canary Islands (1995-1996). Epidemiologic Group of the Canary Society of Endocrinology and Nutrition]**. *Revista clinica espanola* 2000, **200**(5):257-260.

42. Abellana R, Ascaso C, Carrasco JL, Castell C, Tresserras R: **Geographical variability of the incidence of Type 1 diabetes in subjects younger than 30 years in Catalonia, Spain**. *Med Clin (Barc)* 2009, **132**(12):454-458.

43. Goday A, Castell C, Tresserras R, Canela J, Taberner JL, Lloveras G: **Incidence of type 1 (insulin-dependent) diabetes mellitus in Catalonia, Spain. The Catalan Epidemiology Diabetes Study Group**. *Diabetologia* 1992, **35**(3):267-271.

44. Forga L, Goni MJ, Ibanez B, Cambra K, Mozas D, Chueca M: **[Incidence of type 1 diabetes in Navarre, 2009-2012]**. *Anales del sistema sanitario de Navarra* 2014, **37**(2):241-247.

45. Forga L, Goni MJ, Cambra K, Ibanez B, Mozas D, Chueca M, En Representacion del Grupo de Estudio de Diabetes tipo 1 de N: **[Differences by age and gender in the incidence of type 1 diabetes in Navarre, Spain (2009-2011)]**. *Gaceta sanitaria / SESPAS* 2013, **27**(6):537-540.

46. Dahlquist GG, Nystrom L, Patterson CC: **Incidence of type 1 diabetes in Sweden among individuals aged 0-34 years, 1983-2007: an analysis of time trends**. *Diabetes care* 2011, **34**(8):1754-1759.

47. Ostman J, Lonnberg G, Arnqvist HJ, Blohme G, Bolinder J, Ekbom Schnell A, Eriksson JW, Gudbjornsdottir S, Sundkvist G, Nystrom L: **Gender differences and temporal variation in the incidence of type 1 diabetes: results of 8012 cases in the nationwide Diabetes Incidence Study in Sweden 1983-2002**. *Journal of internal medicine* 2008, **263**(4):386-394.

48. Pundziute-Lycka A, Dahlquist G, Nystrom L, Arnqvist H, Bjork E, Blohme G, Bolinder J, Eriksson J, Sundkvist G, Ostman J: **The incidence of Type I diabetes has not increased but shifted to a younger age at diagnosis in the 0-34 years group in Sweden 1983-1998**. *Diabetologia* 2002, **45**:783-791.

49. Nystrom L, Dahlquist G, Ostman J, Wall S, Arnqvist H, Blohme G, Lithner F, Littorin B, Schersten B, Wibell L: **Risk of developing insulin-dependent diabetes mellitus (IDDM) before 35 years of age: indications of climatological determinants for age at onset**. *International journal of epidemiology* 1992, **21**(2):352-358.

50. Blohme G, Nystrom L, Arnqvist HJ, Lithner F, Littorin B, Olsson PO, Schersten B, Wibell L, Ostman J: **Male predominance of type 1 (insulin-dependent) diabetes mellitus in young adults: results from a 5-year prospective nationwide study of the 15-34-year age group in Sweden**. *Diabetologia* 1992, **35**(1):56-62.

51. Thunander M, Petersson C, Jonzon K, Fornander J, Ossiansson B, Torn C, Edvardsson S, Landin-Olsson M: **Incidence of type 1 and type 2 diabetes in adults and children in Kronoberg, Sweden**. *Diabetes research and clinical practice* 2008, **82**(2):247-255.

52. Imkampe AK, Gulliford MC: **Trends in Type 1 diabetes incidence in the UK in 0- to 14-year-olds and in 15- to 34-year-olds, 1991-2008**. *Diabetic medicine : a journal of the British Diabetic Association* 2011, **28**(7):811-814.

53. Bingley PJ, Gale EA: **Incidence of insulin dependent diabetes in England: a study in the Oxford region, 1985-6**. *BMJ* 1989, **298**(6673):558-560.

54. Jordan OW, Lipton RB, Stupnicka E, Cruickshank JK, Fraser HS: **Incidence of type I diabetes in people under 30 years of age in Barbados, West Indies, 1982-1991**. *Diabetes care* 1994, **17**(5):428-431.

55. Legault L, Polychronakos C: **Annual incidence of type 1 diabetes in Quebec between 1989-2000 in children**. *Clin Invest Med* 2006, **29**(1):10-13.

56. Wagenknecht LE, Roseman JM, Herman WH: **Increased incidence of insulin-dependent diabetes mellitus following an epidemic of Coxsackievirus B5**. *American journal of epidemiology* 1991, **133**(10):1024-1031.

57. Wagenknecht LE, Roseman JM, Alexander WJ: **Epidemiology of IDDM in black and white children in Jefferson County, Alabama, 1979-1985**. *Diabetes* 1989, **38**(5):629-633.

58. Vehik K, Hamman RF, Lezotte D, Norris JM, Klingensmith G, Bloch C, Rewers M, Dabelea D: **Increasing Incidence of Type 1 Diabetes in 0- to 17-Year-Old Colorado Youth**. *Diabetes care* 2007, **30**(3):503-509.

59. Kostraba JN, Gay EC, Cai Y, Cruickshanks KJ, Rewers MJ, Klingensmith GJ, Chase HP, Hamman RF: **Incidence of insulin-dependent diabetes mellitus in Colorado**. *Epidemiology* 1992, **3**(3):232-238.

60. Libman IM, LaPorte RE, Becker D, Dorman JS, Drash AL, Kuller L: **Was there an epidemic of diabetes in nonwhite adolescents in Allegheny County, Pennsylvania?** *Diabetes care* 1998, **21**(8):1278-1281.

61. Fishbein HA, Faich GA, Ellis SE: **Incidence and hospitalization patterns of insulin-dependent diabetes mellitus**. *Diabetes care* 1982, **5**(6):630-633.

62. Bell RA, Mayer-Davis EJ, Beyer JW, D'Agostino RB, Jr., Lawrence JM, Linder B, Liu LL, Marcovina SM, Rodriguez BL, Williams D *et al*: **Diabetes in non-Hispanic white youth: prevalence, incidence, and clinical characteristics: the SEARCH for Diabetes in Youth Study**. *Diabetes care* 2009, **32 Suppl 2**:S102-111.

63. Allen C, Palta M, D'Alessio DJ: **Incidence and differences in urban-rural seasonal variation of type 1 (insulin-dependent) diabetes in Wisconsin**. *Diabetologia* 1986, **29**(9):629-633.

64. Gorham ED, Garland FC, Barrett-Connor E, Garland CF, Wingard DL, Pugh WM: **Incidence of insulin-dependent diabetes mellitus in young adults: experience of 1,587,630 US Navy enlisted personnel**. *American journal of epidemiology* 1993, **138**(11):984-987.

65. Tran F, Stone M, Huang CY, Lloyd M, Woodhead HJ, Elliott KD, Crock PA, Howard NJ, Craig ME: **Population-based incidence of diabetes in Australian youth aged 10-18 yr: increase in type 1 diabetes but not type 2 diabetes**. *Pediatric diabetes* 2014, **15**(8):585-590.

66. Sutton DL, Lyle DM, Pierce JP: **Incidence and prevalence of insulin-dependent diabetes mellitus in the zero- to 19-years' age-group in Sydney**. *Med J Aust* 1989, **151**(3):140-141, 144-146.

67. Sasaki A, Okamoto N: **Epidemiology of childhood diabetes in Osaka District, Japan, using the documents from the medical benefits system specific for childhood diabetes**. *Diabetes Res Clin Pract* 1992, **18**(3):191-196.

68. Scott RS, Brown LJ: **Prevalence and incidence of insulin-treated diabetes mellitus in adults in Canterbury, New Zealand**. *Diabetic medicine : a journal of the British Diabetic Association* 1991, **8**(5):443-447.

69. Lin WH, Wang MC, Wang WM, Yang DC, Lam CF, Roan JN, Li CY: **Incidence of and mortality from Type I diabetes in Taiwan from 1999 through 2010: a nationwide cohort study**. *PloS one* 2014, **9**(1):e86172.

70. Washington RE, Orchard TJ, Arena VC, Laporte RE, Tull ES: **Incidence of type 1 and type 2 diabetes in youth in the U.S. Virgin Islands, 2001-2010**. *Pediatric diabetes* 2013, **14**(4):280-287.
