# Supplementary material for: Global epidemiology of type 1 diabetes in young adults and adults: a systematic review
Source: BMC Public Health. 2015 Mar 17;15:255. doi: 10.1186/s12889-015-1591-y (PMC4381393; doi:10.1186/s12889-015-1591-y)
Supplement: Additional file 5: Table S2. — T1D incidences by sex in young adults and adults found in the Systematic Review. Male-to-Female ratios >1 are highlighted. Ref: Reference. First author and publication year in reports of the systematic review using the autoantibodies/C-peptide as diagnosis criteria are highlighted. Inc: incidence per 100.000 persons per year. NW: Nation-wide study. HIGH, LMIC: High, Low-Medium Income Level. (†) Studies used for analyses. (§) The five areas were Ohio (8 counties), Washington State (5 counties), South Carolina, Colorado and California; the table presents the mean incidence calculated retrieved from 5 populations: African American, Asian Pacific Islander, Navajo, Hispanic and non-Hispanic young. Incidence was calculated as the mean of retrieved information: (a) in Jews and other non-Arabs and Arabs; (b) in White and Black populations; (c) in Non-Hispanic Whites and Hispanic Whites. (d) Study giving the total incidence by sex, not by age classes. [file 12889_2015_1591_MOESM5_ESM.docx]

## Additional file 5: Table S2 – T1D incidences by sex in young adults and adults found in the Systematic Review

| **Country, Area** | **First Author, publication year** | **Ref** | **Period** | **Age range** | **Inc. Men** | **Inc. Fem** | **Male/Fem ratio** |
| --- | --- | --- | --- | --- | --- | --- | --- |
| **African Region, LMIC** |  |  |  |  |  |  |  |
| Mauritius: NW | Tuomilehto J., 1993 † | [1] | 1986-1990 | 15-19 | 0.4 | 1.7 | 0.24 |
| **Eastern Mediterranean Region, LMIC** | |  |  |  |  |  |  |
| Iran (Islamic Republic of): Fars | **Pishdad G. R., 2005 †** | [2] | 1990-1994 | 15-29 | 2.7 | 4.0 | 0.68 |
| Libyan Arab Jamahiriya: Benghazi | Kadiki O. A., 1996 † | [3] | 1981-1990 | 15-34 | 14.2 | 9.0 | **1.58** |
| Tunisia: Beja, Monastir, Gafsa | Ben Khalifa F., 1998 † | [4] | 1990-1994 | 15-19 | 6.2 | 8.8 | 0.70 |
| **European Region, LMIC** |  |  |  |  |  |  |  |
| Estonia, NW | Kalits I., 1990 † | [5] | 1988-1988 | 15-39 | 18.2 | 21.9 | 0.83 |
| Lithuania, NW | Ostrauskas R., 2011 † | [6] | 1991-2008 | 15-34 | 10.4 | 6.1 | **1.71** |
| Lithuania, NW | Pundziute-Lycka A., 2003 | [7] | 1991-2000 | 20-39 | 10.2 | 5.4 | **1.89** |
| Lithuania, NW | Ostrauskas R., 2000 | [8] | 1991-1997 | 15-39 | 9.7 | 5.7 | **1.70** |
| Poland: Province of Rzeszow | Sobel-Maruniak A., 2006 | [9] | 1980-1999 | 15-29 | 6.8 | 4.7 | **1.45** |
| Poland: Province of Rzeszow | Grzywa M. A., 1995 † | [10] | 1980-1992 | 15-29 | 6.0 | 5.1 | **1.16** |
| Poland: Warsaw | Wysocki M. J., 1992 † | [11] | 1983-1988 | 15-29 | 6.5 | 4.4 | **1.48** |
| Slovakia: NW | Kyvik K. O., 2004 † | [12] | 1996-1997 | 25-29 | 5.8 | 3.9 | **1.49** |
| **European Region, HIGH** |  |  |  |  |  |  |  |
| Austria: Upper | Rami B., 2001 † | [13] | 1994-1996 | 25-30 | 8.2 | 4.4 | **1.86** |
| Belgium: Antwerp | **Weets I., 2002** † | [14] | 1989-2000 | 15-39 | 7.0 | 10.6 | 0.66 |
| Belgium: Antwerp | **Vandewalle C., 1997** † | [15] | 1989-1995 | 15-39 | 11.0 | 6.9 | **1.59** |
| Bosnia and Herzegovina: Republic of Srpska | Radosevic B., 2013 † | [16] | 1998-2010 | 15-18 | 6.3 | 3.9 | **1.62** |
| Denmark: Copenhagen and Frederiksborg | **Molbak A. G., 1994 †** | [17] | 1973-1977 | >30 | 9.1 | 7.5 | **1.21** |
| Finland: NW | **Lammi N., 2007 †** | [18] | 1992-1996 | 15-39 | 20.1 | 11.8 | **1.70** |
| Israel: NW | Blumenfeld O., 2014 † (a) | [19] | 1997-2010 | 15-17 | 11.0 | 7.4 | **1.49** |
| Israel: NW | Koton S., 2007 | [20] | 1997-2003 | 15-17 | 10.6 | 7.1 | **1.49** |
| Italy: Lombardie | Garancini P., 1991 † | [21] | 1981-1982 | 15-34 | 4.9 | 2.9 | **1.69** |
| Italy: Pavia | Tenconi M. T., 1995 † | [22] | 1988-1992 | 15-29 | 6.9 | 6.9 | **1.00** |
| Italy: Sardinia | Muntoni S., 1992 † | [23] | 1989-1990 | 15-29 | 25.3 | 12.1 | **2.09** |
| Italy: Sardinia (Oristano) | Frongia O., 1997 † | [24] | 1993-1996 | 15-29 | 21.7 | 22.3 | 0.97 |
| Italy: Turin | **Bruno G, 2009 †** | [25] | 2000-2004 | 15-29 | 8.2 | 5.9 | **1.39** |
| Italy: Turin | **Bruno G, 2005 †** | [26] | 1999-2001 | 30-39 | 9.2 | 5.4 | **1.70** |
| Italy: Turin | Bruno G., 1993 | [27] | 1984-1988 | 15-29 | 7.4 | 4.2 | **1.75** |
| Malta: NW | Schranz A. G., 1989 † | [28] | 1980-1987 | 15-24 | 13.3 | 12.2 | **1.09** |
| Netherlands: NW | Ruwaard D., 1994 † | [29] | 1988-1990 | 15-19 | 16.6 | 13.3 | **1.25** |
| Norway: NW | Joner G., 1991 † | [30] | 1978-1982 | 15-29 | 19.0 | 15.0 | **1.27** |
| Slovenia: NW | Radosevic B., 2013 † | [16] | 1998-2010 | 15-18 | 8.4 | 6.6 | **1.27** |
| Spain: Badajoz | Morales-Perez F. M., 2000 † | [31] | 1992-1996 | 15-29 | 10.2 | 7.3 | **1.40** |
| Spain: Canarias Islands | Carrillo Dominguez A., 2000 † | [32] | 1995-1996 | 15-29 | 12.2 | 6.2 | **1.97** |
| Spain: Catalonia | **Abellana R., 2009 †** | [33] | 1989-1998 | 15-29 | 10.6 | 7.0 | **1.51** |
| Spain: Catalonia | Goday A., 1992 | [34] | 1987-1990 | 15-29 | 12.6 | 7.2 | **1.75** |
| Spain: Navarra | **Forga L., 2014** † | [35] | 2009-2012 | 15-29 | 23.0 | 8.2 | **2.80** |
| Spain: Navarra | **Forga L., 2013 †** | [36] | 2009-2011 | 15-29 | 22.4 | 8.3 | **2.69** |
| Sweden: NW | Dahlquist G. G, 2011 † | [37] | 1983-2007 | 15-34 | 18.9 | 9.2 | **2.05** |
| Sweden: NW | Östman J., 2008 | [38] | 1983-2002 | 15-34 | 16.4 | 8.9 | **1.84** |
| Sweden: NW | Pundziute-Lycka A., 2002 | [39] | 1983-1998 | 15-34 | 16.1 | 9.1 | **1.77** |
| Sweden: NW | Nyström L., 1992 | [40] | 1983-1987 | 15-34 | 15.8 | 8.6 | **1.85** |
| Sweden: NW | Blohme G., 1992 | [41] | 1983-1987 | 15-34 | 15.9 | 8.6 | **1.85** |
| Sweden: Kronoberg | **Thunander M., 2008 †** | [42] | 1998-2001 | 20-39 | 21.6 | 9.4 | **2.29** |
| United Kingdom: NW | Imkampe A. K., 2011 † | [43] | 1991-2008 | 15-34 | 16.5 | 8.5 | **1.94** |
| United Kingdom: Oxford region | Bingley P. J., 1989 | [44] | 1985-1986 | 15-19 | 16.0 | 16.5 | 0.97 |
| **Region of the Americas, HIGH** |  |  |  |  |  |  |  |
| United States of America: Alabama (Jefferson County) | Wagenknecht L. E., 1989 (b) | [45] | 1979-1985 | 15-19 | 8.6 | 10.0 | 0.86 |
| United States of America: Colorado | Vehik K., 2007 † (c) | [46] | 2000-2004 | 15-17 | 10.7 | 8.2 | **1.31** |
| United States of America: Colorado | Kostraba J. N., 1992 | [47] | 1978-1988 | 15-17 | 9.8 | 7.6 | **1.30** |
| United States of America: five areas § | **Bell R., 2009 †** | [48] | 2002-2005 | 15-19 | 8.3 | 7.7 | **1.08** |
| United States of America: Wisconsin | Allen C., 1986 † | [49] | 1970-1979 | 15-29 | 11.7 | 8.3 | **1.41** |
| United States of America: The United States Navy | Gorham C., 1993 (b) | [50] | 1974-1988 | 17-34 | 24.4 | 24.9 | 0.98 |
| **Western Pacific Region, HIGH** |  |  |  |  |  |  |  |
| Japan: Osaka | Sasaki A., 1992 † | [51] | 1978-1988 | 15-18 | 1.8 | 1.5 | **1.21** |
| New Zealand: Canterbury | Scott, R. S., 1991† | [52] | 1981-1986 | 20-39 | 11.4 | 7.3 | **1.56** |
| **Other Regions currently non-WHO** |  |  |  |  |  |  |  |
| Taiwan: NW | **Lin W. -H., 2013 †** | [53] | 1999-2010 | 15-29 | 3.2 | 4.1 | 0.77 |
| *No available information* |  |  |  |  |  |  |  |
| **African Region, LMIC** |  |  |  |  |  |  |  |
| United Republic of Tanzania: Dar es Salaam | Swai A. B., 1993 † | [54] | 1982-1991 | NA | NA | NA | NA |
| **European Region, LMIC** |  |  |  |  |  |  |  |
| Croatia: Zagreb | Roglic G., 1995 † | [55] | 1988-1992 | NA | NA | NA | NA |
| Poland: Bialystok | Kretowski A., 2001 † | [56] | 1994-1998 | NA | NA | NA | NA |
| Romania: Bucharest | Ionescu-Tirgoviste C., 1994 † | [57] | 1981-1991 | NA | NA | NA | NA |
| **European Region, HIGH** |  |  |  |  |  |  |  |
| Belgium: Antwerp | **Weets I., 2007 †** | [58] | 1989-2003 | NA | NA | NA | NA |
| France: Aquitaine, Lorraine, Basse Normandie, Haute Normandie | Charkaluk M. L., 2002 † | [59] | 1988-1997 | NA | NA | NA | NA |
| France: Aquitaine, Lorraine, Haute-Normandie, and Basse-Normandie. | Levy-Marchal C., 1998 | [60] | 1988-1995 | NA | NA | NA | NA |
| Israel: NW | Sella T., 2010 (d) | [61] | 2000-2008 | NA | NA | NA | NA |
| Luxembourg: NW | De Beaufort C. E., 1988 † (d) | [62] | 1977-1986 | NA | NA | NA | NA |
| **Region of the Americas, HIGH** |  |  |  |  |  |  |  |
| Canada: Quebec | Legault L., 2006 † | [63] | 2000 | NA | NA | NA | NA |
| United States of America: Alabama (Jefferson County) | Wagenknecht L. E., 1991 † (d) | [64] | 1979-1988 | NA | NA | NA | NA |
| United States of America: Pennsylvania (Allegheny) | Libman I. M., 1998 † (d) | [65] | 1990-1994 | NA | NA | NA | NA |
| United States of America: Rhode Island | Fishbein H. A., 1982 † (d) | [66] | 1979-1980 | NA | NA | NA | NA |
| **Region of the Americas, LMIC** |  |  |  |  |  |  |  |
| Barbados: NW | Jordan O. W., 1994 † (d) | [67] | 1982-1991 | NA | NA | NA | NA |
| **Western Pacific Region, HIGH** |  |  |  |  |  |  |  |
| Australia: New South Wales | Tran F., 2014 † | [68] | 2001-2008 | NA | NA | NA | NA |
| Australia: Sydney (Southern Metropolitan Heath Region) | Sutton L., 1989 † | [69] | 1984-1987 | NA | NA | NA | NA |
| **Other Regions currently non-WHO** |  |  |  |  |  |  |  |
| US Virgin Islands: NW | Washington R. E., 2013 † | [70] | 2001-2010 | NA | NA | NA | NA |

**Male-to-Female ratios >1** are highlighted. **Ref**: Reference. First author and publication year in reports of the systematic review using the autoantibodies/C-peptide as diagnosis criteria are highlighted. **Inc**: incidence per 100.000 persons per year. **NW**: Nation-wide study. **HIGH, LMIC**: High, Low-Medium Income Level. **(†)** Studies used for analyses. **(§)** The five areas were Ohio (8 counties), Washington State (5 counties), South Carolina, Colorado and California; the table presents the mean incidence calculated retrieved from 5 populations: African American, Asian Pacific Islander, Navajo, Hispanic and non-Hispanic young. Incidence was calculated as the mean of retrieved information: (**a**) in Jews and other non-Arabs and Arabs; **(b)** in White and Black populations; **(c)** in Non-Hispanic Whites and Hispanic Whites. **(d)** Study giving the total incidence by sex, not by age classes.

**References**

1. Tuomilehto J, Dabee J, Karvonen M, Dowse GK, Gareeboo H, Virtala E, Tiihonen M, Alberti KG, Zimmet PZ: **Incidence of IDDM in Mauritian children and adolescents from 1986 to 1990**. *Diabetes care* 1993, **16**(12):1588-1591.

2. Pishdad GR: **Low incidence of type 1 diabetes in Iran**. *Diabetes care* 2005, **28**(4):927-928.

3. Kadiki OA, Reddy MR, Marzouk AA: **Incidence of insulin-dependent diabetes (IDDM) and non-insulin-dependent diabetes (NIDDM) (0-34 years at onset) in Benghazi, Libya**. *Diabetes research and clinical practice* 1996, **32**(3):165-173.

4. Ben Khalifa F, Mekaouar A, Taktak S, Hamhoum M, Jebara H, Kodia A, Zouari B, Chakroun M: **A five-year study of the incidence of insulin-dependent diabetes mellitus in young Tunisians (preliminary results)**. *Diabetes Metab* 1997, **23**(5):395-401.

5. Kalits I, Podar T: **Incidence and prevalence of type 1 (insulin-dependent) diabetes in Estonia in 1988**. *Diabetologia* 1990, **33**(6):346-349.

6. Ostrauskas R, Zalinkevicius R, Jurgeviciene N, Radzeviciene L, Lasaite L: **The incidence of type 1 diabetes mellitus among 15-34 years aged Lithuanian population: 18-year incidence study based on prospective databases**. *BMC public health* 2011, **11**:813.

7. Pundziute-Lycka A, Urbonaite B, Ostrauskas R, Zalinkevicius R, Dahlquist GG: **Incidence of type 1 diabetes in Lithuanians aged 0-39 years varies by the urban-rural setting, and the time change differs for men and women during 1991-2000**. *Diabetes care* 2003, **26**(3):671-676.

8. Ostrauskas R, Zalinkevicius R: **Incidence in young adulthood-onset Type 1 diabetes mellitus in Lithuania during 1991-1997. Lithuanian Epidemiology Diabetes Study Group**. *Diabetes, nutrition & metabolism* 2000, **13**(2):68-74.

9. Sobel-Maruniak A, Grzywa M, Orlowska-Florek R, Staniszewski A: **The rising incidence of type 1 diabetes in south-eastern Poland. A study of the 0-29 year-old age group, 1980-1999**. *Endokrynol Pol* 2006, **57**(2):127-130.

10. Grzywa MA, Sobel AK: **Incidence of IDDM in the province of Rzeszow, Poland, 0- to 29-year-old age-group, 1980-1992**. *Diabetes care* 1995, **18**(4):542-544.

11. Wysocki MJ, Chanska M, Bak M, Czyzyk AS: **Incidence of insulin-dependent diabetes mellitus in Warsaw, Poland, in children and young adults, 1983-1988**. *World Health Stat Q* 1992, **45**(4):315-320.

12. Kyvik KO, Nystrom L, Gorus F, Songini M, Oestman J, Castell C, Green A, Guyrus E, Ionescu-Tirgoviste C, McKinney PA *et al*: **The epidemiology of Type 1 diabetes mellitus is not the same in young adults as in children**. *Diabetologia* 2004, **47**(3):377-384.

13. Rami B, Waldhor T, Schober E: **Incidence of Type I diabetes mellitus in children and young adults in the province of Upper Austria, 1994-1996**. *Diabetologia* 2001, **44 Suppl 3**:B45-47.

14. Weets I, De Leeuw I, Du Caju M, Rooman R, Keymeulen B, Mathieu C, Rottiers R, Daubresse J, Rocour-Brumioul D, Pipeleers D *et al*: **The incidence of type 1 diabetes in the age group 0-39 years has not increased in Antwerp (Belgium) between 1989 and 2000: evidence for earlier disease manifestation**. *Diabetes Care* 2002, **25**:840-846.

15. Vandewalle CL, Coeckelberghs MI, De Leeuw IH, Du Caju MV, Schuit FC, Pipeleers DG, Gorus FK: **Epidemiology, clinical aspects, and biology of IDDM patients under age 40 years. Comparison of data from Antwerp with complete ascertainment with data from Belgium with 40% ascertainment. The Belgian Diabetes Registry**. *Diabetes care* 1997, **20**(10):1556-1561.

16. Radosevic B, Bukara-Radujkovic G, Miljkovic V, Pejicic S, Bratina N, Battelino T: **The incidence of type 1 diabetes in Republic of Srpska (Bosnia and Herzegovina) and Slovenia in the period 1998-2010**. *Pediatric diabetes* 2013, **14**(4):273-279.

17. Molbak AG, Christau B, Marner B, Borch-Johnsen K, Nerup J: **Incidence of insulin-dependent diabetes mellitus in age groups over 30 years in Denmark**. *Diabetic medicine : a journal of the British Diabetic Association* 1994, **11**(7):650-655.

18. Lammi N, Taskinen O, Moltchanova E, Notkola IL, Eriksson JG, Tuomilehto J, Karvonen M: **A high incidence of type 1 diabetes and an alarming increase in the incidence of type 2 diabetes among young adults in Finland between 1992 and 1996.** *Diabetologia* 2007, **50**(7):1393-1400.

19. Blumenfeld O, Dichtiar R, Shohat T, Israel IRSG: **Trends in the incidence of type 1 diabetes among Jews and Arabs in Israel**. *Pediatric diabetes* 2014, **15**(6):422-427.

20. Koton S: **Incidence of type 1 diabetes mellitus in the 0- to 17-yr-old Israel population, 1997-2003**. *Pediatric diabetes* 2007, **8**(2):60-66.

21. Garancini P, Gallus G, Calori G, Formigaro F, Micossi P: **Incidence and prevalence rates of diabetes mellitus in Italy from routine data: a methodological assessment**. *European journal of epidemiology* 1991, **7**(1):55-63.

22. Tenconi MT, Devoti G, Albani I, Lorini R, Martinetti M, Fratino P, Ferrari E, Ferrero E, Severi F: **IDDM in the province of Pavia, Italy, from a population-based registry.A descriptive study**. *Diabetes care* 1995, **18**(7):1017-1019.

23. Muntoni S, Songini M: **High incidence rate of IDDM in Sardinia. Sardinian Collaborative Group for Epidemiology of IDDM**. *Diabetes care* 1992, **15**(10):1317-1322.

24. Frongia O, Mastinu F, Sechi GM: **Prevalence and 4-year incidence of insulin-dependent diabetes mellitus in the province of Oristano (Sardinia, Italy)**. *Acta Diabetol* 1997, **34**(3):199-205.

25. Bruno G, Novelli G, Panero F, Perotto M, Monasterolo F, Bona G, Perino A, Rabbone I, Cavallo-Perin P, Cerutti F: **The incidence of type 1 diabetes is increasing in both children and young adults in Northern Italy: 1984–2004 temporal trends**. *Diabetologia* 2009, **52**(12):2531-2535.

26. Bruno G, Runzo C, Cavallo-Perin P, Merletti F, Rivetti M, Pinach S, Novelli G, Trovati M, Cerutti F, Pagano G: **Incidence of type 1 and type 2 diabetes in adults aged 30-49 years: the population-based registry in the province of Turin, Italy**. *Diabetes care* 2005, **28**(11):2613-2619.

27. Bruno G, Merletti F, Vuolo A, Pisu E, Giorio M, Pagano G: **Sex differences in incidence of IDDM in age-group 15-29 yr. Higher risk in males in Province of Turin, Italy**. *Diabetes care* 1993, **16**(1):133-136.

28. Schranz AG, Prikatsky V: **Type 1 diabetes in the Maltese Islands**. *Diabetic medicine : a journal of the British Diabetic Association* 1989, **6**(3):228-231.

29. Ruwaard D, Hirasing RA, Reeser HM, van Buuren S, Bakker K, Heine RJ, Geerdink RA, Bruining GJ, Vaandrager GJ, Verloove-Vanhorick SP: **Increasing incidence of type I diabetes in The Netherlands. The second nationwide study among children under 20 years of age**. *Diabetes care* 1994, **17**(6):599-601.

30. Joner G, Sovik O: **The incidence of type 1 (insulin-dependent) diabetes mellitus 15-29 years in Norway 1978-1982**. *Diabetologia* 1991, **34**(4):271-274.

31. Morales-Perez FM, Barquero-Romero J, Perez-Miranda M: **Incidence of type I diabetes among children and young adults (0-29 years) in the province of Badajoz, Spain during 1992 to 1996**. *Acta Paediatr* 2000, **89**(1):101-104.

32. Carrillo Dominguez A: **[Incidence of type 1 diabetes mellitus in the Canary Islands (1995-1996). Epidemiologic Group of the Canary Society of Endocrinology and Nutrition]**. *Revista clinica espanola* 2000, **200**(5):257-260.

33. Abellana R, Ascaso C, Carrasco JL, Castell C, Tresserras R: **Geographical variability of the incidence of Type 1 diabetes in subjects younger than 30 years in Catalonia, Spain**. *Med Clin (Barc)* 2009, **132**(12):454-458.

34. Goday A, Castell C, Tresserras R, Canela J, Taberner JL, Lloveras G: **Incidence of type 1 (insulin-dependent) diabetes mellitus in Catalonia, Spain. The Catalan Epidemiology Diabetes Study Group**. *Diabetologia* 1992, **35**(3):267-271.

35. Forga L, Goni MJ, Ibanez B, Cambra K, Mozas D, Chueca M: **[Incidence of type 1 diabetes in Navarre, 2009-2012]**. *Anales del sistema sanitario de Navarra* 2014, **37**(2):241-247.

36. Forga L, Goni MJ, Cambra K, Ibanez B, Mozas D, Chueca M, En Representacion del Grupo de Estudio de Diabetes tipo 1 de N: **[Differences by age and gender in the incidence of type 1 diabetes in Navarre, Spain (2009-2011)]**. *Gaceta sanitaria / SESPAS* 2013, **27**(6):537-540.

37. Dahlquist GG, Nystrom L, Patterson CC: **Incidence of type 1 diabetes in Sweden among individuals aged 0-34 years, 1983-2007: an analysis of time trends**. *Diabetes care* 2011, **34**(8):1754-1759.

38. Ostman J, Lonnberg G, Arnqvist HJ, Blohme G, Bolinder J, Ekbom Schnell A, Eriksson JW, Gudbjornsdottir S, Sundkvist G, Nystrom L: **Gender differences and temporal variation in the incidence of type 1 diabetes: results of 8012 cases in the nationwide Diabetes Incidence Study in Sweden 1983-2002**. *Journal of internal medicine* 2008, **263**(4):386-394.

39. Pundziute-Lycka A, Dahlquist G, Nystrom L, Arnqvist H, Bjork E, Blohme G, Bolinder J, Eriksson J, Sundkvist G, Ostman J: **The incidence of Type I diabetes has not increased but shifted to a younger age at diagnosis in the 0-34 years group in Sweden 1983-1998**. *Diabetologia* 2002, **45**:783-791.

40. Nystrom L, Dahlquist G, Ostman J, Wall S, Arnqvist H, Blohme G, Lithner F, Littorin B, Schersten B, Wibell L: **Risk of developing insulin-dependent diabetes mellitus (IDDM) before 35 years of age: indications of climatological determinants for age at onset**. *International journal of epidemiology* 1992, **21**(2):352-358.

41. Blohme G, Nystrom L, Arnqvist HJ, Lithner F, Littorin B, Olsson PO, Schersten B, Wibell L, Ostman J: **Male predominance of type 1 (insulin-dependent) diabetes mellitus in young adults: results from a 5-year prospective nationwide study of the 15-34-year age group in Sweden**. *Diabetologia* 1992, **35**(1):56-62.

42. Thunander M, Petersson C, Jonzon K, Fornander J, Ossiansson B, Torn C, Edvardsson S, Landin-Olsson M: **Incidence of type 1 and type 2 diabetes in adults and children in Kronoberg, Sweden**. *Diabetes research and clinical practice* 2008, **82**(2):247-255.

43. Imkampe AK, Gulliford MC: **Trends in Type 1 diabetes incidence in the UK in 0- to 14-year-olds and in 15- to 34-year-olds, 1991-2008**. *Diabetic medicine : a journal of the British Diabetic Association* 2011, **28**(7):811-814.

44. Bingley PJ, Gale EA: **Incidence of insulin dependent diabetes in England: a study in the Oxford region, 1985-6**. *BMJ* 1989, **298**(6673):558-560.

45. Wagenknecht LE, Roseman JM, Alexander WJ: **Epidemiology of IDDM in black and white children in Jefferson County, Alabama, 1979-1985**. *Diabetes* 1989, **38**(5):629-633.

46. Vehik K, Hamman RF, Lezotte D, Norris JM, Klingensmith G, Bloch C, Rewers M, Dabelea D: **Increasing Incidence of Type 1 Diabetes in 0- to 17-Year-Old Colorado Youth**. *Diabetes care* 2007, **30**(3):503-509.

47. Kostraba JN, Gay EC, Cai Y, Cruickshanks KJ, Rewers MJ, Klingensmith GJ, Chase HP, Hamman RF: **Incidence of insulin-dependent diabetes mellitus in Colorado**. *Epidemiology* 1992, **3**(3):232-238.

48. Bell RA, Mayer-Davis EJ, Beyer JW, D'Agostino RB, Jr., Lawrence JM, Linder B, Liu LL, Marcovina SM, Rodriguez BL, Williams D *et al*: **Diabetes in non-Hispanic white youth: prevalence, incidence, and clinical characteristics: the SEARCH for Diabetes in Youth Study**. *Diabetes care* 2009, **32 Suppl 2**:S102-111.

49. Allen C, Palta M, D'Alessio DJ: **Incidence and differences in urban-rural seasonal variation of type 1 (insulin-dependent) diabetes in Wisconsin**. *Diabetologia* 1986, **29**(9):629-633.

50. Gorham ED, Garland FC, Barrett-Connor E, Garland CF, Wingard DL, Pugh WM: **Incidence of insulin-dependent diabetes mellitus in young adults: experience of 1,587,630 US Navy enlisted personnel**. *American journal of epidemiology* 1993, **138**(11):984-987.

51. Sasaki A, Okamoto N: **Epidemiology of childhood diabetes in Osaka District, Japan, using the documents from the medical benefits system specific for childhood diabetes**. *Diabetes Res Clin Pract* 1992, **18**(3):191-196.

52. Scott RS, Brown LJ: **Prevalence and incidence of insulin-treated diabetes mellitus in adults in Canterbury, New Zealand**. *Diabetic medicine : a journal of the British Diabetic Association* 1991, **8**(5):443-447.

53. Lin WH, Wang MC, Wang WM, Yang DC, Lam CF, Roan JN, Li CY: **Incidence of and mortality from Type I diabetes in Taiwan from 1999 through 2010: a nationwide cohort study**. *PloS one* 2014, **9**(1):e86172.

54. Swai AB, Lutale JL, McLarty DG: **Prospective study of incidence of juvenile diabetes mellitus over 10 years in Dar es Salaam, Tanzania**. *BMJ* 1993, **306**(6892):1570-1572.

55. Roglic G, Pavlic-Renar I, Sestan-Crnek S, Prasek M, Kadrnka-Lovrencic M, Radica A, Metelko Z: **Incidence of IDDM during 1988-1992 in Zagreb, Croatia**. *Diabetologia* 1995, **38**(5):550-554.

56. Kretowski A, Kowalska I, Peczynska J, Urban M, Green A, Kinalska I: **The large increase in incidence of Type I diabetes mellitus in Poland**. *Diabetologia* 2001, **44 Suppl 3**:B48-50.

57. Ionescu-Tirgoviste C, Paterache E, Cheta D, Farcasiu E, Serafinceanu C, Mincu I: **Epidemiology of diabetes in Bucharest**. *Diabetic medicine : a journal of the British Diabetic Association* 1994, **11**(4):413-417.

58. Weets I, Rooman R, Coeckelberghs M, De Block C, Van Gaal L, Kaufman JM, Keymeulen B, Mathieu C, Weber E, Pipeleers DG *et al*: **The age at diagnosis of type 1 diabetes continues to decrease in Belgian boys but not in girls: a 15-year survey**. *Diabetes Metab Res Rev* 2007, **23**(8):637-643.

59. Charkaluk ML, Czernichow P, Levy-Marchal C: **Incidence data of childhood-onset type I diabetes in France during 1988-1997: the case for a shift toward younger age at onset**. *Pediatr Res* 2002, **52**(6):859-862.

60. Levy-Marchal C: **[Evolution of the incidence of IDDM in childhood in France]**. *Revue d'epidemiologie et de sante publique* 1998, **46**(3):157-163.

61. Sella T, Shoshan A, Goren I, Shalev V, Blumenfeld O, Laron Z, Chodick G: **A retrospective study of the incidence of diagnosed Type 1 diabetes among children and adolescents in a large health organization in Israel, 2000-2008**. *Diabetic medicine : a journal of the British Diabetic Association* 2011, **28**(1):48-53.

62. de Beaufort CE, Michel G, Glaesener G: **The incidence of type 1 (insulin-dependent) diabetes mellitus in subjects aged 0-19 years in Luxembourg: a retrospective study from 1977 to 1986**. *Diabetologia* 1988, **31**(10):758-761.

63. Legault L, Polychronakos C: **Annual incidence of type 1 diabetes in Quebec between 1989-2000 in children**. *Clin Invest Med* 2006, **29**(1):10-13.

64. Wagenknecht LE, Roseman JM, Herman WH: **Increased incidence of insulin-dependent diabetes mellitus following an epidemic of Coxsackievirus B5**. *American journal of epidemiology* 1991, **133**(10):1024-1031.

65. Libman IM, LaPorte RE, Becker D, Dorman JS, Drash AL, Kuller L: **Was there an epidemic of diabetes in nonwhite adolescents in Allegheny County, Pennsylvania?** *Diabetes care* 1998, **21**(8):1278-1281.

66. Fishbein HA, Faich GA, Ellis SE: **Incidence and hospitalization patterns of insulin-dependent diabetes mellitus**. *Diabetes care* 1982, **5**(6):630-633.

67. Jordan OW, Lipton RB, Stupnicka E, Cruickshank JK, Fraser HS: **Incidence of type I diabetes in people under 30 years of age in Barbados, West Indies, 1982-1991**. *Diabetes care* 1994, **17**(5):428-431.

68. Tran F, Stone M, Huang CY, Lloyd M, Woodhead HJ, Elliott KD, Crock PA, Howard NJ, Craig ME: **Population-based incidence of diabetes in Australian youth aged 10-18 yr: increase in type 1 diabetes but not type 2 diabetes**. *Pediatric diabetes* 2014, **15**(8):585-590.

69. Sutton DL, Lyle DM, Pierce JP: **Incidence and prevalence of insulin-dependent diabetes mellitus in the zero- to 19-years' age-group in Sydney**. *Med J Aust* 1989, **151**(3):140-141, 144-146.

70. Washington RE, Orchard TJ, Arena VC, Laporte RE, Tull ES: **Incidence of type 1 and type 2 diabetes in youth in the U.S. Virgin Islands, 2001-2010**. *Pediatric diabetes* 2013, **14**(4):280-287.
